# In vitro assessment of bacterial supernatants on hypothalamic gene expression: implications for appetite regulation

**DOI:** 10.1038/s41522-025-00820-9

**Published:** 2025-10-03

**Authors:** Cristina Cuesta-Marti, Benjamin Valderrama, Thomaz Bastiaanssen, John F. Cryan, Catherine Stanton, Siobhain M. O’Mahony, Gerard Clarke, Harriët Schellekens

**Affiliations:** 1 Department of Anatomy and Neuroscience, University College Cork, Cork, Ireland; 2 APC Microbiome Ireland, University College Cork, Cork, Ireland; 3 Teagasc Food Research Centre, Moorepark, Fermoy Co, Cork, Ireland; 4 Department of Psychiatry and Neurobehavioural Science, University College Cork, Cork, Ireland

**Keywords:** Biological techniques, Microbiology, Anatomy

## Abstract

Bacterial metabolites, such as short-chain fatty acids (SCFAs), influence energy balance, appetite, and endocrine function. Investigating cell-free (CFSs) and cell-free conditioned supernatants (CCSs) containing SCFAs and other microbial metabolites may help unravel the mechanisms underpinning these potential benefits for metabolic health. This study evaluated the neuroactive potential of two bacterial species, *Bifidobacterium longum* APC1472 and *Limosilactobacillus reuteri* ATCC PTA 6475, known for their metabolic health benefits. In silico analysis predicted the capacity of these bacteria to produce neuroactive metabolites involved in gut-brain communication. Next, untargeted metabolomics was used to evaluate the predicted functional capability of these two species to produce metabolites under different growth conditions. CFSs and CCSs were tested on embryonic and adult mouse hypothalamic cells to assess their effects on appetite-regulating gene expression. Results revealed supernatant type- and species-specific metabolite profiles, identifying *B. longum* APC1472 and *L. reuteri* ATCC PTA 6475 as acetate producers, with *B. longum* APC1472 also identified as a tryptophan producer. The distinct metabolite profiles of CFSs and CCSs from these two species induced specific effects on the modulation of ghrelin receptor and glucagon-like receptor 1 gene expression in hypothalamic cells. These findings validate an in vitro approach to identify bacterial metabolites with potential neuroactive and metabolic health benefits, demonstrated through modulation of mouse hypothalamic gene expression.

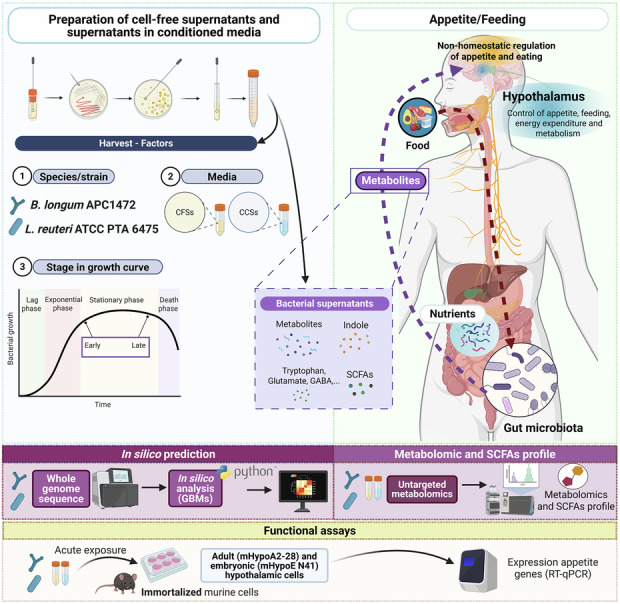

## Introduction

The last decade has seen an enormous expansion of microbiome research, demonstrating its importance across a broad spectrum of human health^1,2^. The human microbiome is described as a collection of microorganisms including bacteria, viruses, fungi, and archaea in and on the human body^2^. Furthermore, the gut microbiota is a key actor in the bidirectional communication between the gut and the brain, also described as the microbiota-gut-brain axis, involving neuro-immune-endocrine pathways^2^. The gut microbiota is able to process dietary fibers otherwise indigestible by the host, and produce bacteria-derived metabolites, such as short-chain fatty acids (SCFAs), hormones, neurotransmitters, or bile acids^2,3^. These metabolites can impact host physiology, brain function, and host behavior, either directly via signaling through the vagus nerve or indirectly via the systemic circulation after crossing the blood-brain barrier (BBB)^4–6^.

Among the different roles of the gut microbiota in host health, research is focusing on its involvement in the maintenance of host energy and metabolic homeostasis and metabolic and mental health^7–9^. This is not surprising since metabolic disorders often are associated with altered food intake or eating behavior (e.g. obesity, diabetes mellitus, metabolic syndrome, or binge eating) and are increasing in incidence worldwide^10,11^. Unhealthy eating is a major contributor to non-communicable diseases, including metabolic disorders such as diabetes or obesity, as well as cardiovascular disorders and cancer (WHO)^11,12^. Metabolic and eating disorders are linked to chronic alterations in energy balance and food intake^10,13^. Food intake is regulated by homeostatic processes (i.e. appetite/hunger) as well as non-homeostatic processes (i.e. motivation to eat, food pleasure or food reward, and liking, learning, memory, and cognitive aspects of feeding)^14^. Growing evidence suggests the gut microbiota as a major contributor to metabolic health^7,15^, homeostatic feeding as well as hedonic eating^16,17^. Microbiota-targeted interventions, including post-, pre-, pro- or synbiotics, are gaining attention as potential treatments for obesity and other metabolic disorders^7,15,18^.

The arcuate nucleus (ARC) of the hypothalamus, expressing the orexigenic agouti-related protein (AgRP)/neuropeptide Y (NPY) neurons and the anorexigenic pro-opiomelanocortin (POMC)/cocaine- and amphetamine-regulated transcript (CART) neurons^19^, is the key central region that governs the homeostatic control of appetite and food intake, via key neuropeptides including ghrelin (GHRL), glucagon-like peptide 1 (GLP-1), cholecystokinin (CCK), peptide YY (PYY), leptin, among others^14,20,21^.

Recent evidence highlights a role for the gut microbiota in the modulation of appetite and food intake via modulation of hypothalamic signaling^8,17,22^. It is suggested that specific bacteria-derived metabolites from diet and other non-dietary components (including caseinolytic peptidase B protein (ClpB), muramyl dipeptide (MDP), or lipopolysaccharide (LPS)) can mediate these effects on appetite and food intake via enteroendocrine, immune and humoral signaling, which are converged on the hypothalamic centers of homeostatic regulation of food intake^16,22–24^. For example, the *Hafnia alvei* HA4597 strain was shown to generate the ClpB protein, which led to suppress food intake, reduce fat mass gain, and body weight in obese and hyperphagic ob/ob mice^22^. Translationally, this strain increased a feeling of fullness, led to weight loss, and a reduction in circumference in overweight subjects on moderate hypocaloric diet^25^. According to the International Scientific Association of Probiotics and Prebiotics (ISAPP), bacteria-derived metabolites and compounds are substances such as SCFAs, or bacteriocins, synthesized by microorganisms and can be found within the microbial cell or secreted into the extracellular environment^26,27^, and can be generated under laboratory conditions and collected in cell-free supernatants (CFSs) for the interrogative screening using functional cellular assays^28,29^.

In this study, we characterize the metabolic activity of *Bifidobacterium longum* strain APC1472, a bacteria isolated from feces of a 1-week-old breastfed infant, and its potential to produce metabolites that influence the expression of host genes important for appetite regulation^30,31^. We have previously shown that administration of *B. longum* APC1472 induced positive anti-obesity effects in HFD-induced obese mice, which were partially translated following evaluation in overweight and obese individuals^15^. Interestingly, *B. longum* APC1472 induced changes of in the hypothalamic expression of neuropeptides involved in appetite regulation in C57BL/6 mice, including CART and POMC^15^, and to attenuate enduring alterations in eating behavior induced by an early-life exposure to a high-fat/high-sugar diet in adult female and male mice (under review). However, the mechanisms through which this strain exerts its effects remain unclear, particularly whether its effects on hypothalamic neuropeptide expression are mediated via the neuroactive metabolites it produces. Therefore, in the present study, we characterized the predicted capacity of *B. longum* APC1472 to produce metabolites involved in gut-brain axis communication based on its genome^32^, using an in silico approach, and subsequently analyzed the metabolite profile, including SCFAs, of CFSs and bacterial cell-free conditioned supernatants (CCSs).

We compared the metabolite profile of *B. longum* APC1472 and the commercial bacteria *Limosilactobacillus reuteri* ATCC PTA 6475. *B. longum* APC1472 was previously shown to prevent diet-induced obesity and obesity-associated inflammation in mice^33,34^, and to modulate the expression and signaling of the anorexigenic neuropeptide oxytocin in the hypothalamus of mice^34–36^. While *L. reuteri* ATCC PTA 6475 has been shown to impact metabolic health^33^, the role of this bacteria on the central regulation of appetite has never been studied to our knowledge. Also, *L. reuteri* ATCC PTA 6475 has been shown to display anti-microbial^37^, immune-modulatory^38^, or antioxidant^39^ and to impact on gut-brain signaling including metabolic and behavioral alterations, such as social behavior in mice^35,40^ and children^41^.

Bacteria-derived metabolites, which have previously been shown to impact on appetite regulation^42^, are suggested to be able to cross both the intestinal epithelial barrier^43–45^ and the BBB^5,42^. In the present study, we assessed the impact of the cell-free supernatants from these two species, as a model to specifically assess the effect of bacteria-derived metabolites in the absence of bacterial cells or their components, on the expression of genes related to appetite regulation pathways, namely GHRL, ghrelin receptor or growth hormone secretagogue receptor (GHSR), GLP-1 receptor (GLP-1R) and NPY receptor type 1 (NPY1R), in hypothalamic cells.

## Results

### In silico prediction for synthesis or degradation of neuroactive compounds by *B. longum* APC1472 and *L. reuteri* ATCC PTA 6475

**Fig. 1:**
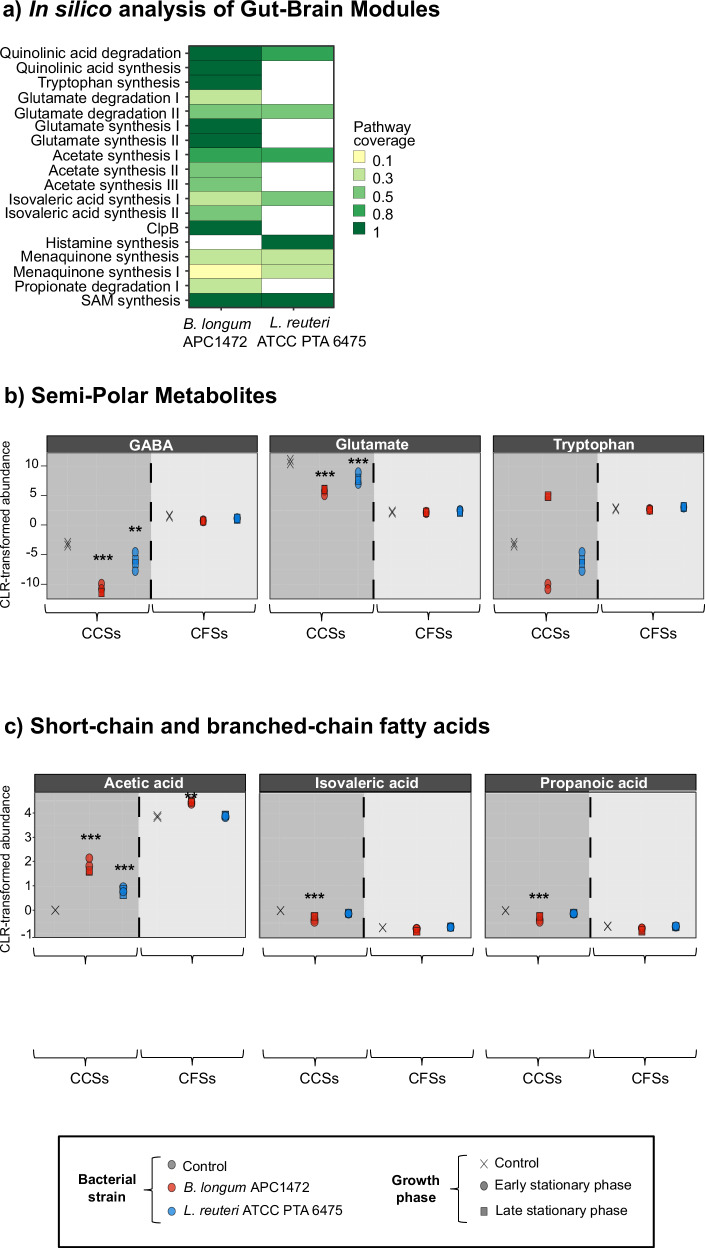
Gut-brain modules from *B. longum* APC1472 and *L. reuteri* ATCC PTA 6475 and validation for the synthesis/degradation via semi-polar metabolite profiling and short-chain fatty acids and branched-chain fatty acids of the cell-free supernatants and cell-free conditioned supernatants from these two species. **a** Coverage of AQ6 gut-brain modules (GBMs) detected in the genomes of *B. longum* APC1472 and *L. reuteri* ATCC PTA 6475. Cut-off values indicate the completion of the entire metabolic pathway that was found in the genome (value of 1 indicating the identification of the complete pathway while 0.1 indicates the identification of ≤10% of the enzymes in that pathway). Raw abundance of **(b**) semi-polar metabolites and (**c**) acetate, propionate, and isovaleric acid in CFSs or CCSs from *B. longum* APC1472 and *L. reuteri* ATCC PTA 6475 when grown in mMRS or Buffer B medias, respectively, compared to control (*N* = 4 group/media combining both time points or growth phases). CFSs cell-free supernatants, CCSs cell-free conditioned supernatants, ClpB caseinolytic peptidase B protein, CLR centered log-ratio, GABA γ-aminobutyric acid, SAM S-adenosylmethionine. Significant differences assessed by ANOVA followed by Tukey-adjusted post-hoc comparisons (*metabolite* ~ *bacterial* *specie*s + *media*) (*p* = *p*.adjusted value, p* < 0.05, **p < 0.01, ***p < 0.001).

We first identified the neuroactive potential to modulate the gut-brain axis of the bacteria *B. longum* APC1472 and *L. reuteri* ATCC PTA 6475, which was inferred from their genomic content by assessing the presence of the gut-brain modules (GBMs)^32^. Figure 1a shows the list of pathways for the biosynthesis or degradation of neuroactive compounds with the coverage of the genes that *B. longum* APC1472 and *L. reuteri* ATCC PTA 6475 possess to encode for the partial or complete pathway based on their genomes.

The in silico analysis showed that *B. longum* APC1472 has a potential to fulfil the pathway to synthesize quinolinic acid, tryptophan, glutamate, S-adenosyilmethionine (SAM) and ClpB as well as to degrade quinolinic acid (Fig. 1a). Regarding the SCFAs and branched-chain fatty acids (BCFAs), *B. longum* APC1472 encodes for the majority of genes necessary for pathway to synthesize acetate and isovaleric acid, and to degrade propionate in lower coverage (Fig. 1a). *L. reuteri* ATCC PTA 6475 has a potential to fulfil the pathway to also synthesize SAM as well as histamine (Fig. 1a). *L. reuteri* ATCC PTA 6475 encodes for the majority of the pathway required for the degradation of quinolinic acid and the synthesis of the SCFA acetate, and with less coverage, for the degradation of glutamate and synthesis of isovaleric acid (Fig. 1a).

### Metabolomic analysis of neuroactive compounds present in supernatants from *B. longum* APC1472 and *L. reuteri* ATCC PTA 6475

Next, we sought to validate that the metabolic pathways identified by in silico means were used by the bacteria in in vitro settings via metabolomic profiling of the CFSs and CCSs from *B. longum* APC1472 and *L. reuteri* ATCC PTA 6475. Interestingly, we identified that the raw abundance of the metabolites and SCFAs present in the CCSs from *B. longum* APC1472 and *L. reuteri* ATCC PTA 6475 was low but above the limit of detection (Fig. 1b, c). Therefore, we investigated the metabolite composition and SCFAs profile of both bacterial supernatant types, as well as their impact on the expression of hypothalamic genes involved in appetite regulation using in vitro assays. Focusing on the relative abundance, while no differences were observed in the relative abundance of GABA and glutamate in CFSs of both *B. longum* APC1472 and *L. reuteri* ATCC PTA 6475 compared to the relative to the abundance of the average metabolite in control CFSs, CCSs of *B. longum* APC1472 and *L. reuteri* ATCC PTA 6475 had significant lower relative abundance of GABA (*B. longum* APC1472, (F_(2, 14)_ = 37.04, *p* = 5.95 × 10^-8^); *L. reuteri* ATCC PTA 6475, (F_(2, 14)_ = 37.04, *p* = 0.0029)) and glutamate (*B. longum* APC1472, (F_(2, 14)_ = 38.58, *p* = 6.55 × 10^−8^); *L. reuteri* ATCC PTA 6475, (F_(2, 14)_ = 38.58, *p* = 0.00005)) compared to control CCSs (Fig. 1b). Interestingly, CCSs of *B. longum* APC1472 showed different relative abundance of tryptophan depending on the growth phase, with lower relative abundance at early stationary phase compared to control and higher abundance at late stationary phase (media effect F(1, 14) = 14.47, *p* = 0.002, *p*.adjusted = 0.003), bacteria effect (F_(2, 14)_ = 0.29, *p*.value = 0.752, *p*.adjusted = 0.782), interaction media:bacteria (F_(2, 14)_ = 0.47, *p*.value = 0.634, *p*.adjusted = 0.678) (Fig. 1b). Noteworthy, the statistical testing for growth phase effects was not performed due to our study design and the lack of significant differences in supernatant dissimilarity between growth phases. No significant differences were observed in the relative abundance of tryptophan in CCSs of *L. reuteri* ATCC PTA 6475 or CFSs of *B. longum* APC1472 and *L. reuteri* ATCC PTA 6475 compared to the respective control (Fig. 1b). CCSs of *B. longum* APC1472 and *L. reuteri* ATCC PTA 6475 had significant higher relative abundance of the SCFA acetate compared to control CCSs (*B. longum* APC1472, F_(2, 14)_ = 24.9, *p* = 6.96 × 10^−9^); *L. reuteri* ATCC PTA 6475, F_(2, 14)_ = 24.9, *p* = 0.000125)), whereas only CFSs of *B. longum* APC1472 had a significant higher relative abundance of acetate compared to control CFSs (F_(2, 14)_ = 24.9, *p* = 0.0025) (Fig. 1c). CCSs of *B. longum* APC1472 presented a significant lower relative abundance of the SCFA propionic acid, also known as propionate (F_(2, 14)_ = 3.08, *p* = 0.00042), as well as of the BCFA isovaleric acid (also known as 3-methylbutanoic acid or β-methyl butyric acid or IVA) (F_(2, 14)_ = 4.46, *p* = 0.00038) compared to control, while no differences were observed in CFSs of *B. longum* APC1472 or CCSs nor CFSs of *L. reuteri* ATCC PTA 6475 (Fig. 1c).

As observed in Supplementary Fig. S3, CCSs of *L. reuteri* ATCC PTA 6475 (F_(2, 14)_ = 94.33, *p*.adjusted = 5.95 × 10^−^^11^) and *B. longum* APC1472 (F_(2, 14)_ = 94.33, *p*.adjusted = 8.18 × 10^−^^8^) presented significantly higher relative abundance of histamine compared to control, with a significant interaction between bacteria and media (F_(2, 14)_ = 94.33, *p*.value = 7.51 × 10^−9^, *p*.adjusted = 3.13 × 10^−^^8^), and significant main effects of bacteria (F_(2, 14)_ = 147.22, *p*.value = 3.97 × 10^−10^, *p*.adjusted = 2.12 × 10^−9^) and media (F_(1, 14)_ = 197.48, *p*.value = 1.20 × 10^−9^, *p*.adjusted = 5.76 × 10^−9^). Additionally, no significant differences were observed in the relative abundance of SAM in CFSs and CCSs of *B. longum* APC1472 or *L. reuteri* ATCC PTA 6475 compared to the respective control (Supplementary Fig. S3).

Effects of media, bacteria, as well as statistical differences in the rest of metabolites detected with confidence level 1 and 2a in the supernatants above the limit of detection can be found in https://github.com/Benjamin-Valderrama/cuesta2025_probiotic_appetite/tree/main/outputs.

### Impact of bacterial species, media and growth stage on the production of metabolites produced in vitro by *B. longum* APC1472 and *L. reuteri* ATCC PTA 6475

**Fig. 2:**
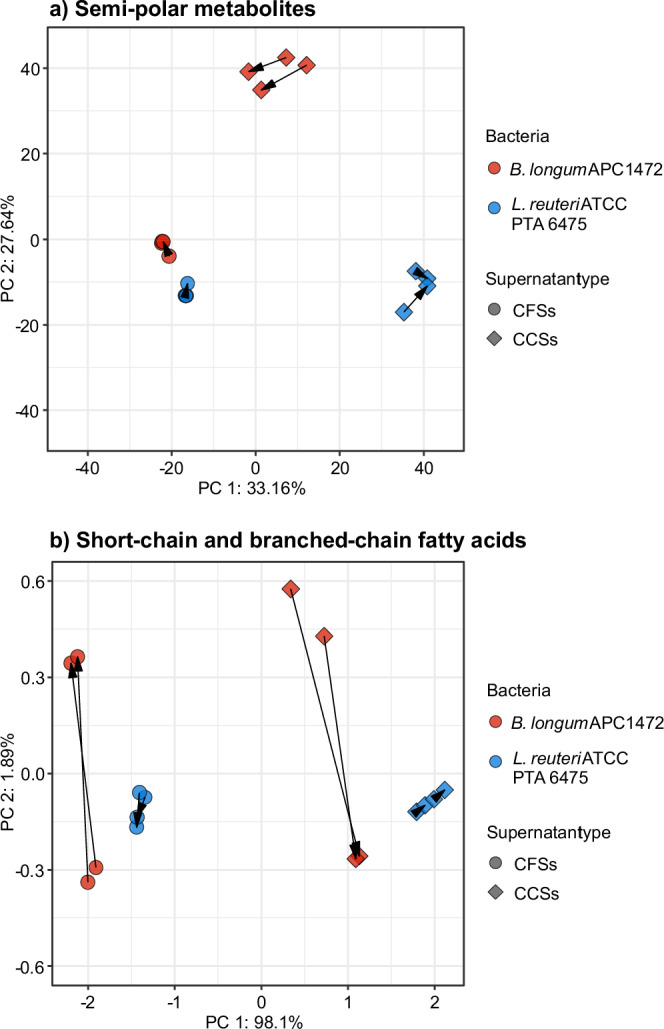
Principal Component Analysis plot of the overall composition of the cell-free supernatants and cell-free conditioned supernatants from *B. longum* APC1472 and *L. reuteri* ATCC PTA 6475 harvested at early or late stationary phase. **a** Overall composition of supernatants analyzing semipolar metabolites. **b** Overall supernatant composition analyzing SCFAs and BCFA. **a**, **b** *N* = 2 group/media/growth phases (*N* = 4 group/media combining both time points or growth phases). Arrow connects CFS samples from the same combination of growth media and bacteria over time, with the origin of the arrow indicating the early stationary phase and the head of the arrow the late stationary phase. CFSs cell-free supernatants, CCSs cell-free conditioned supernatants.

Next, we conducted a Principal Component Analysis (PCA) of both the semi-polar metabolites and SCFAs/BCFAs present in the CFSs and CCSs from *B. longum* APC1472 and *L. reuteri* ATCC PTA 6475 (Fig. 2a, b), to further investigate the impact of species, media (growth or conditioned assay media), and growth phase on the profile of these bacteria-derived metabolites. Interestingly, we found large differences in the profile of semi-polar metabolites between CCSs and CFSs of both *B. longum* APC1472 and *L. reuteri* ATCC PTA 6475 (PERMANOVA: media (supernatant type) effect, (Pseudo F_(1, 10)_ = 21.65, *R*² = 0.293, *p* = 0.0001)) (Fig. 2a). Moreover, large significant differences were also observed in the composition of semi-polar metabolites present in CFSs between species, with a much larger separation than within CFSs (PERMANOVA: Bacteria effect, (Pseudo F_(2, 10)_ = 10.77, *R*² = 0.292, *p* = 0.0001); interaction media:bacteria, (Pseudo F_(2, 10)_ = 7.05, *R*² = 0.191, *p* = 0.0001)) (Fig. 2a). No significant differences were observed in the profile of semi-polar metabolites between growth phases in CFSs and CCSs of both *B. longum* APC1472 and *L. reuteri* ATCC PTA 6475 (PERMANOVA: growth phase effect, (Pseudo F_(1, 10)_ = 1.57, *R*² = 0.021, *p* = 0.161); interaction media:growth phase, (Pseudo F_(1, 10)_ = 1.73, *R*² = 0.023, *p* = 0.127); interaction bacteria:growth phase, (Pseudo F_(1, 10)_ = 1.61, *R*² = 0.022, *p* = 0.141); interaction media:bacteria:growth phase (Pseudo F_(1, 10)_ = 1.55, *R*² = 0.021, *p* = 0.161)) (Fig. 2a).

Focusing on the composition of SCFAs and BCFAs, no significant differences were found between bacterial species or between media types in CFSs and CCSs of *B. longum* APC1472 and *L. reuteri* ATCC PTA 6475 (PERMANOVA: bacteria effect, (Pseudo F_(2, 10)_ = 1.20, *R*² = 0.114, *p* = 0.355); media effect, (Pseudo F(1, 10) = 0.41, *R*² = 0.021, p = 0.544); interaction media:bacteria, (Pseudo F_(2, 10)_ = 0.027, *R*² = 0.027, *p* = 0.986)) (Fig. 2b). However, a significant interaction between media and growth phase was observed (interaction media: growth phase, (Pseudo F(1, 10) = 6.01, *R*² = 0.305, *p* = 0.035), while no significant effects were found for growth phase alone or other interactions (growth phase/time effect, (Pseudo F_(1, 10)_ = 0.14, *R*² = 0.007, *p* = 0.739); interaction bacteria: growth phase, (Pseudo F(1, 10) = 0.61, *R*² = 0.031, *p* = 0.442); interaction bacteria:growth phase, (Pseudo F_(1, 10)_ = 0.613, *R*² = 0.031, *p* = 0.442); interaction media:bacteria:growth phase (Pseudo F_(1, 10)_ = 0.24, *R*² = 0.012, *p* = 0.639)) (Fig. 2b). These results highlight the effects of different factors on the composition of bacterial-derived metabolites, mainly impacted by the media.

### Differences in chemical class of metabolites present in supernatants from *B. longum* APC1472 and *L. reuteri* ATCC PTA 6475 according to bacterial species and media

**Fig. 3:**
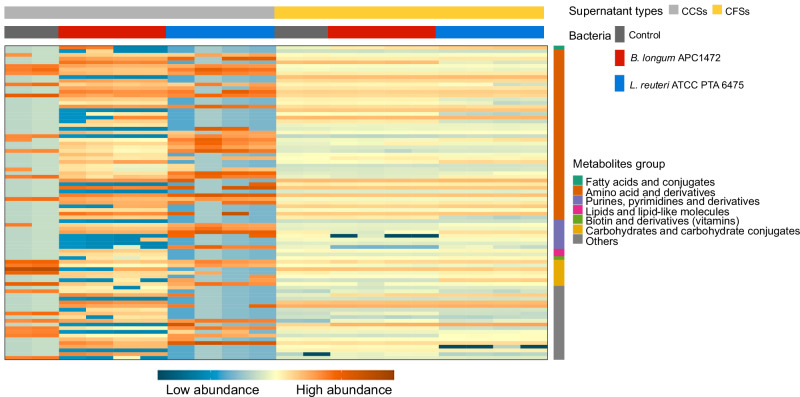
Heatmap showing the abundance of semi-polar metabolites present in the cell-free supernatants and cell-free conditioned supernatants from *B. longum* APC1472 and *L. reuteri* ATCC PTA 6475 categorized by chemical classification. *N* = 4 group/media combining both time points or growth phases. Heatmap represents CLR-transformed abundance of all semipolar metabolites.For each metabolite, its abundance was standardized across the different experimental groups using Z-scores. CFSs cell-free supernatants, CCSs cell-free conditioned supernatants.

To further investigate the profile present in the bacteria-derived metabolites of CFSs and CCSs, we categorized these semi-polar metabolites according to the corresponding chemical class. The metabolic profile of CCSs and CFSs from *B. longum* APC1472 and *L. reuteri* ATCC PTA 6475 exhibited distinct patterns across multiple chemical classes when compared to respective control media (Fig. 3).

CCSs from both *B. longum* APC1472 and *L. reuteri* ATCC PTA 6475 demonstrated a lower relative abundance of fatty acids and conjugates compared to the control. In contrast, CFSs from both species showed an elevated relative abundance of these metabolites, particularly in the case of *L. reuteri* ATCC PTA 6475, where the increase was more pronounced (Fig. 3). CCSs from both species displayed a higher relative abundance of metabolites from the amino acids and conjugates, and purines, pyrimidines, and derivatives classes compared to the control, with *L. reuteri* showing a stronger increase. However, this tendency was not observed in CFSs of *B. longum* nor *L. reuteri*, where no significant differences in the relative abundance of these metabolite classes compared to control were observed. The analysis of purines, pyrimidines, and derivatives revealed a higher relative abundance in CCSs from both species, again more pronounced in *L. reuteri*. In contrast, CFSs from *B. longum* APC1472 exhibited a lower relative abundance of these metabolites compared to control, while CFSs from *L. reuteri* showed no significant differences compared to control. CCSs from *B. longum* APC1472 showed an increased relative abundance of lipids, lipid-like molecules, and biotin derivatives, while CCSs from *L. reuteri* had a reduced relative abundance of these metabolites compared to control. Notably, no significant differences in these classes were observed in CFSs from either species relative to the control. The relative abundance of carbohydrates and their conjugates was higher in CCSs from *B. longum* APC1472, whereas CCSs from *L. reuteri* ATCC PTA 6475 had a lower abundance compared to control. In the CFSs, *L. reuteri* showed a slight increase in the relative abundance of this class of metabolites, while CFSs from *B. longum* APC1472 exhibited no major changes compared to control. Differences in the relative abundance of metabolites from other chemical classes were more pronounced in CCSs compared to CFSs. In general, CCSs exhibited larger differences when comparing to control across various classes, with differences between species also being evident. In summary, *B. longum* APC1472 and *L. reuteri* ATCC PTA 6475 display differential metabolite profile in both CCSs and CFSs, with more significant changes observed in CCSs across most chemical classes compared to CFSs (Fig. 3). These differences highlight the distinct metabolic contributions of each species depending on environmental conditions such as standard growth media vs conditioned assay media, with higher differences between species observed in the chemical class of metabolites present in supernatants in conditioned assay media.

### Supernatants from *B. longum* APC1472 and *L. reuteri* ATCC PTA 6475 modulate hypothalamic expression of genes involved in appetite regulation

To further investigate the mechanisms by which the administration of *B. longum* APC1472 induces complex changes in central gene expression in the host and whether these effects are mediated by bacteria-derived metabolites, we incubated CFSs and CCSs from *B. longum* APC1472 to embryonic mHypoE-N41 and adult mHypoA2/28 hypothalamic mouse cells for 4 h. We studied the impact of this acute exposure of the supernatants containing microbial-derived metabolites in the expression of *Ghrl*, *Ghsr*, *Glp1r,* and *Npy1r*, genes involved in appetite regulation^14,20,21,46^, and to play a role in the microbiota-gut-brain axis^15,29,42,47–49^, in hypothalamic cell lines. We also investigated the effect of supernatants from another bacterial species, commercially available and broadly studied, the *L. reuteri* ATCC PTA 6475. For both species, supernatants were harvested in ideal mMRS media (CFSs) or later conditioned in conditioned media (Buffer B, CCSs), and each one at two points in the growth’s phase, early or late stationary phase (ESP, and LSP, respectively). Growth’s curves of *B. longum* APC1472 and *L. reuteri* ATCC PTA 6475 in mMRS media are shown in Supplementary Fig. S4a and S4b respectively.

**Fig. 4:**
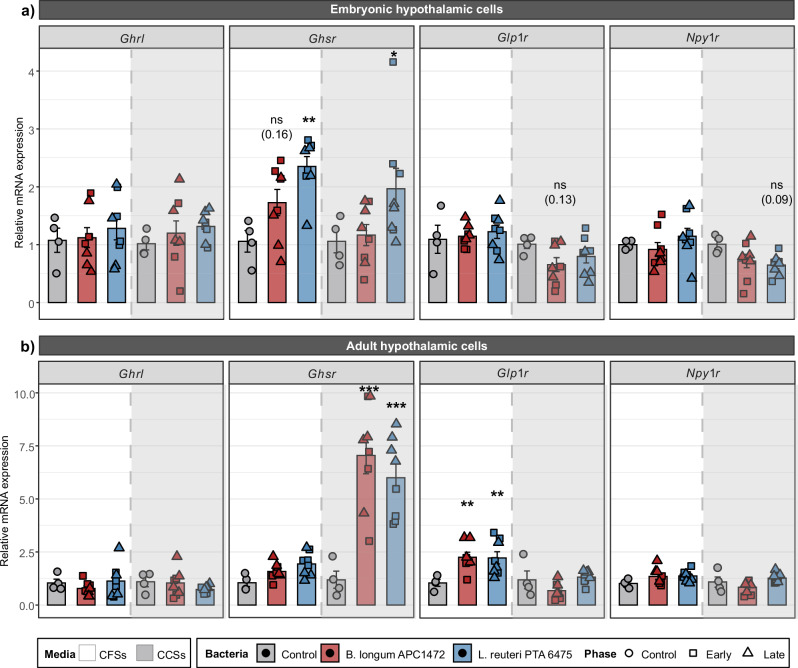
Effects of acute exposure of cell-free supernatants and cell-free conditioned supernatants from *B. longum* APC1472 and *L. reuteri* ATCC PTA 6475 on the expression on genes involved in appetite regulation in embryonic and adulthypothalamic mouse cells. **a**, **b** Relative mRNA expression of the orexigenic marker ghrelin (*Ghrl*) and its receptor (growth hormone secretagogue-receptor, *Ghsr*), glucagon-like peptide 1 receptor (*Glp1r*), and the receptor type 1 of orexigenic neuropeptide Y (*Npy1r*) after acute exposure of cell-free supernatants (CFSs, white background) and cell-free conditioned supernatants (CCSs, gray background) from *B. longum* APC1472 or *L. reuteri* ATCC PTA 6475 to (**a**) embryonic mHypoE-N41 and (**b**) adult mHypoA2/28 hypothalamic mouse cells. CFSs and CCSs from *B. longum* APC1472 or *L. reuteri* ATCC PTA 6475 were harvested either at early stationary phase (early) or late stationary phase (late). **a**, **b** *N* = 4 group (bacteria in both growth phases)/media. Data are shown as mean ± SEM. Data are significantly different (*p < 0.05, **p < 0.01, ***p < 0.001) according to two-way ANOVA followed by Dunnett’s contrasts. CFSs cell-free supernatants, CCSs cell-free conditioned supernatants.

MTT assay was used as a cytotoxicity assay to test cell viability when exposing different dilutions with the CFSs or CCSs of both species (Supplementary Fig. S6). Positive control for cell death, a dilution 1/10 of CFSs media control (*p* = 0.007), a 1/3 dilution of CFSs of both species for both embryonic mHypoE-N41 (*p* < 0.001) (Supplementary Fig. S6a) and adult mHypoA2/28 hypothalamic mouse cells (*p* < 0.001) (Supplementary Fig. S6b) and a 1/2 dilution of CCSs for adult mHypoA2/28 cells (*p* < 0.001) (Supplementary Fig. S6d) were cytotoxic. For this reason, a dilution 1/10 of CFSs and 1/3 of CCSs was selected for the in vitro assays. Exposure to the selected dilution of CFSs and CCSs from *B. longum* APC1472 and *L. reuteri* ATCC PTA 6475 was shown to not alter the cell viability of embryonic and adult hypothalamic cells (Supplementary Fig. S6a–S6d, and further statistical data in Supplementary Table S2). Here, we showed that CFSs and CCSs of *L. reuteri* ATCC PTA 6475 significantly increased the expression of ghrelin receptor (*Ghsr*) in embryonic mHypoE-N41 cells (Fig. 4a, and Supplementary Table S3 for all the statistical data including main effects). Interestingly, only CCSs of *B. longum* APC1472 and *L. reuteri* ATCC PTA 6475 increased significantly the expression of *Ghsr* in mHypoA2/28 cells (Fig. 4b). In addition, only CFSs from *B. longum* APC1472 and *L. reuteri* ATCC PTA 6475 significantly upregulated the expression of glucagon-like peptide 1 (GLP-1) receptor (*Glp1r*) in adult mHypoA2/28 cells (Fig. 4b). No differences were observed in *Glp1r* expression by the exposure of CFSs or CCSs of *B. longum* APC1472 or *L. reuteri* ATCC PTA 6475 in embryonic mHypoE-N41 cells (Fig. 4a), nor by CCSs of these two species in adult mHypoA2/28 cells (Fig. 4b). No significant differences were found by the exposure of the CFSs or CCSs supernatants from *B. longum* APC1472 or *L. reuteri* ATCC PTA 6475 on the expression of the orexigenic peptide ghrelin (*Ghrl*) or of the receptor of the orexigenic Npy (*Npy1r*) in either embryonic mHypoE-N41 cells or adult mHypoA2/28 hypothalamic cells (Fig. 4a, b). Overall, these findings show that metabolite-containing bacterial supernatants from *B. longum* APC1472 and *L. reuteri* ATCC PTA 6475 modulate the expression of the genes *Ghsr* and *Glp1r*, involved in appetite regulation in hypothalamic cells. Moreover, these findings suggest specie-specific effects of the metabolite-containing bacterial supernatants depending on environmental-specific metabolic production including media and growth phase.

## Discussion

Accumulating findings are highlighting the involvement of the microbiota-gut-brain axis in regulating host appetite and eating behavior^16,50^. Research has shown that both the endogenous gut microbiota as well as commercially available probiotics can produce metabolites derived from the host diet, such as SCFAs, hormones, neurotransmitters, and other non-dietary components (including ClpB, MDP, or LPS) that can influence appetite and eating behavior^16,42,47,51,52^. Some of these metabolites can reach the brain and exert those effects either directly by entering the systemic circulation, cross the BBB, or via signaling through the vagus nerve^6,53,54^. In our study, we developed a comprehensive workflow that starts with in silico predictions, followed by validation through metabolomic analysis, and culminates in the assessment of the putative functional effects of bacteria-derived metabolites from two bacterial species using cellular assays to investigate their potential effects on hypothalamic cell lines to modulate the expression of key genes involved in host appetite regulation. We modified the published framework capturing curated microbial pathways involved in metabolism and gut-brain communication by the gut microbiota^32^, to assess whether the single bacteria *B. longum* APC1472 and *L. reuteri* ATCC PTA 6475 partially or fully encode the genes required for the synthesis or degradation of neuroactive compounds. We then analyzed the composition of semi-polar metabolites as well as SCFAs and BCFAs present in CFSs and CCSs of *B. longum* APC1472 and *L. reuteri* ATCC PTA 6475 to further validate the in silico analysis. While previous research has characterized the metabolite composition of CFSs of different bacterial species, including *L. reuteri* strains^55^, this is the first study, to our knowledge, to analyze the metabolite profile of CFSs from *L. reuteri* ATCC PTA 6472, which is one of the most investigated and commercially available *L. reuteri* strain. Moreover, the metabolite profile of *B. longum* APC1472 supernatants has not previously been documented.

In the present study, we analyzed the relative (using centered log-ratio (CLR)) abundance of the metabolites and SCFAs/BCFAs present in CFSs and CCSs of *B. longum* APC1472 and *L. reuteri* ATCC PTA 6475. While CLR transformation is a useful approach for comparing relative abundances of metabolites, it is important to interpret these data alongside the raw absolute abundance data. This is particularly critical because metabolites present at very low absolute concentrations in the samples can appear proportionally higher in CLR-transformed relative abundance data, potentially impacting on the interpretation about the functional significance. While we observed that the raw absolute abundance of some metabolites, including SCFAs and BCFAs, present in CCSs was low, this low raw absolute abundance was still above the limit of detection confirming their presence in the CCSs samples (Supplementary Fig. S2 and Fig. 1b). Importantly, the biological activity of metabolites in vivo requires different concentration ranges. For instance, signaling of these metabolites at the gut epithelium or vagal terminals may occur at nanomolar or micromolar levels^56,57^. Similarly, low concentrations of SCFAs have been reported to cross the BBB^42^. SCFAs can also exert biological effects at nanomolar to micromolar concentrations (1–100 μM) via activation of specific G-protein coupled receptors (GPCRs), such as GPCR-41, GPCR-43^6,58^.

While GBMs in silico analysis found that neither *B. longum* APC1472 nor *L. reuteri* ATCC PTA 6475 encode for the biosynthetic pathway to produce or degrade the neuroactive compound GABA (Fig. 1a), we identified a reduction in its relative abundance in CCSs of *B. longum* APC1472 and *L. reuteri* ATCC PTA 6475 compared to control CCSs (Fig. 1b). These findings may suggest that these two species could potentially have consumed this metabolite. Importantly, GABA is an inhibitory neurotransmitter that orchestrates food intake and energy balance by modulating the activity of hypothalamic neurons responsible for hunger and satiety signaling^59^. Our findings may suggest that these bacteria may be transporting GABA or using it as source of carbon and nitrogen^60^, though the physiological impact may be limited to local gut-brain axis signaling rather than direct CNS effects due to its limited ability to effectively cross the BBB^61^. While the genes for GABA transport and shunting are characterized for some bacteria such as *E. coli* or *L. monocytogenes*^62–64^, the GBMs in silico analysis performed do not cover their prediction.

While no significant differences were observed in the relative abundance of tryptophan in the supernatants from *L. reuteri* ATCC PTA 6475, we observed a higher relative abundance of tryptophan in CCSs of *B. longum* APC1472 at late stationary phase while lower relative abundance in the CCSs at early stationary phase (Fig. 1b). These findings align with the in silico analysis of GBMs demonstrated that *B. longum* APC1472 encode for the complete biosynthetic pathway to produce tryptophan (Fig. 1a). This also aligns with previous studies showing that certain bacteria produce specific metabolites, such as GABA or reuterin, depending on the growth phases^65,66^.

*Bifidobacterium* and lactic acid bacteria, mainly *Limosilactobacillus reuteri*, among other bacteria have been shown to catabolize tryptophan^67,68^. It has been recently shown that the genome of a *Lactiplantibacillus plantarum* strain (LRCC-5314) encodes almost the complete biosynthetic pathway for tryptophan synthesis^69^. Tryptophan metabolism, which can be directly or indirectly regulated by the gut microbiota, impacts host physiology and physiopathology^3,70^. Tryptophan is a precursor of serotonin, which is a neurotransmitter involved in the regulation of stress response, satiety, and mood^70,71^. A dysregulation in tryptophan metabolism has been observed in certain pathologies including inflammatory bowel diseases, irritable bowel syndrome, metabolic disorders, infectious diseases, and neuropsychiatric disorders^70^. Indole, a metabolite derived from tryptophan metabolism by the gut microbiota, has been shown to stimulate the production of the incretin GLP-1 in enteroendocrine L cells and its secretion^49,70^. Diets with low tryptophan have been linked to reduction of food intake^72,73^, potentially due to alterations in serotonin levels. In contrast, higher dietary tryptophan has been shown to upregulate *Ghsr* expression in fundus (stomach) and increase circulating ghrelin levels, which was associated with an increase in food intake and body weight in pigs^74^. Tryptophan administration has also been recently shown to counteract the effects of chronic mild stress-induced binge eating on the hypothalamic expression of appetite markers, including AgRP, orexin receptor type 1 (OX1R), which was translated in vitro on hypothalamic cells^71^. Tryptophan metabolism plays a crucial role in appetite regulation through multiple mechanisms, including kynurenine pathway, as well as synthesis and signaling of serotonin and ghrelin.

In our study, we have also shown, using the in silico pipeline of the GBMs, that the genome of *B. longum* APC1472 encodes most of the genes required for the synthesis of acetate (Fig. 1a). Targeted metabolomic analysis of SCFAs confirmed that both CCSs and CFSs of *B. longum* APC1472 have higher relative abundance of acetate compared to control, with higher increases in CCSs (Fig. 1c). In silico analysis demonstrated that *L. reuteri* ATCC PTA 6475 encodes the majority of components required for acetate synthesis (Fig. 1a). Metabolomics results showed a significant increase in the relative abundance of acetate in CCSs of *L. reuteri* ATCC PTA 6475 compared to control CCSs, while no differences were observed in CFSs compared to control (Fig. 1c). To our knowledge, only one other study has evaluated the production of acetate by *L. reuteri* ATCC PTA 6475. Pallin et al. 2016, identified acetate, lactate and ethanol in samples from heat-treated barley flour fermented with *L. reuteri* ATCC PTA 6475, even though in lower abundance than in samples fermented with other *L. reuteri* strains^75^. As further reviewed elsewhere^6^, *Bifidobacterium adolescentis*^76^ and members of the Bacteroidetes phylum^6,77,78^ had been shown to produce the SCFA acetate, a bacteria-derived metabolite that impacts multiple host biological processes such as metabolic, immune intestinal, and cardiovascular functions^6,57,79^. Frost et al. 2014 showed that colonic acetate can cross the BBB in mice, and an intraperitoneal injection of acetate reduced appetite and food intake accompanied by an upregulation of *Pomc* and downregulation of *Agrp* expression in the ARC of the hypothalamus, without altering circulating levels of GLP-1 or PYY^42^. These results suggest a potential direct role of acetate on orexigenic and anorexigenic signaling in the ARC of the hypothalamus, the main center in control of appetite regulation.

In the present work, we have shown that CFSs and CCSs of *B. longum* APC1472 and *L. reuteri* ATCC PTA 6475 have indeed different metabolite profiles (including differences in chemical classes), including SCFAs composition (Figs. 2–3). Furthermore, these distinct profiles of metabolites, including the SCFAs, from bacterial supernatants from *B. longum* APC1472 and *L. reuteri* ATCC PTA 6475 induced specific changes in hypothalamic cell lines in the expression of different genes involved in appetite regulation in the host, mainly ghrelin receptor (*Ghsr*) and *Glp1r* (Fig. 4). The effects of *B. longum* APC1472 and *L. reuteri* ATCC PTA 6475 supernatants on *Ghsr* and *Glp1r* gene expression in embryonic and adult hypothalamic cells depended on the bacterial species and supernatant type (CFSs or CCSs).

While the modulation of hypothalamic expression of *Ghsr* and *Glp1r* by supernatants from *B. longum* APC1472 and *L. reuteri* ATCC PTA 6475 may be potentially mediated by acetate and tryptophan as major contributors, we only observed a higher relative abundance of tryptophan in supernatants from *B. longum* APC1472 harvested at late stationary phase but CCSs of both *B. longum* and *L. reuteri* ATCC PTA 6475 had higher relative abundance of acetate (Fig. 1c). An administration of *B. longum* APC1472 was previously shown to upregulate significantly the expression of the orexigenic peptide *Ghrl* in the hypothalamus of HFD-fed mice and reduced fasting glucose in the healthy overweight and obese individuals but did not alter blood markers of satiety including total and active ghrelin or GLP-1, PYY and leptin levels^15^. CCSs from *B. longum* APC1472 were also previously shown to significantly decreased the basal levels of GHSR-1a internalization in transfected Hek293a-GHSR-1a–EGFP cells with similar efficacy as the inverse agonist [D-Arg1, D-Phe5, D-Trp7,9, Leu11]-substance P, and CCSs from *B. longum* APC1472 also inhibited *ghrelin*-mediated GHSR-1a internalization in the same cells^29^. These findings suggest the presence of higher number of GHSR available for signaling in the transfected Hek cells after exposure to CCSs from *B. longum* APC1472, aligning with our current findings demonstrating the upregulation of *Ghsr* expression in adult hypothalamic cells. In the same study, we previously showed that exposure of Hek293a-*GHSR*-1a–EGFP to CCSs from *B. longum* APC1472 increased ghrelin-induced phospho-ERK levels compared to ghrelin alone^29^. While these previous findings in Hek cells support the functional relevance of the observed transcriptional changes, the current study is limited to mRNA-level measurements in hypothalamic cells. Further validation at the protein and receptor levels as well as signaling pathway, will be important to confirm these effects in a hypothalamic context and should be explored in future studies.

While the administration of *B. longum* APC1472 did not alter the levels of fecal SCFAs, including acetate, in the individuals^15^, SCFAs levels were not analyzed in the fecal, caecal or blood samples from the mice supplemented with this strain^15^. In the present study, we have shown that *B. longum* APC1472 is an acetate producer as observed by the higher relative abundance of acetate present in supernatant derived from this bacteria (Fig. 1c). Thus, suggesting that the previous and current findings on modulating ghrelinergic signaling by supernatants derived from *B. longum* APC1472 may be mediated by the derived metabolite SCFA acetate.

It is important to highlight that other bacteria-derived metabolites or compounds present in the supernatants could also be mediating those effects. Other potential bacteria-derived compounds that could be contributing to the effects exerted by the bacterial supernatants are S-adenosylmethionine (SAM) and histamine, since in silico evaluation of GBMs revealed that both bacterial species possess the capability to synthesize SAM and histamine in the case of *L. reuteri* ATCC PTA 6475 (Fig. 1a). Previous findings showed that SAM reduced body weight gain, enhanced insulin sensitivity and mitochondrial function in diabetic rats^80^ while also showing a positive association with adiposity in obese individuals^81^, suggesting a role in metabolic regulation and energy homeostasis. Metabolomic analysis did not identify a significant increase in SAM relative abundance in the supernatants from *L. reuteri* ATCC PTA 6475 or *B. longum* APC1472 (Supplementary Fig. S3). *L. reuteri* ATCC PTA 6475 has previously been shown to produce other compounds such as reuterin and histamine^66,82^. Central histamine is produced in the hypothalamus, and it has been extensively shown that it displays an anorexigenic effect that suppresses food intake directly via histamine H1-receptors or indirectly by mediation of other anorexigenic signals such as leptin^83^. Our findings suggest that the neuroactive metabolite histamine, found elevated in CCSs of *L. reuteri* ATCC PTA 6475 as well as of *B. longum* APC1472 (Supplementary Fig. S3), likely contributes to the observed in vitro modulation of hypothalamic gene expression related to appetite regulation pathways.

Noteworthy, our study examined the metabolite profile, including SCFAs/BCFAs, in the CFSs and CCSs of *B. longum* APC1472 and *L. reuteri* ATCC PTA 6475, with a focus on the comprehensive analysis of small molecules and/or metabolites, but did not analyze proteins or other compounds, such as lipidic compounds. The effects exerted by the bacterial supernatants from *B. longum* APC1472 on the expression of genes related to appetite regulation pathways in hypothalamic cells could also be contributed by other bacterial-derived compounds, such as ClpB, in line with the GBMs analysis identifying that this bacteria encodes the ClpB gene (Fig. 1a). ClpB is a bacterial protein mimetic of α-MSH, which has been recently suggested to be involved in signaling satiety^84,85^ and to impact on body weight, fat mass, and meal frequency^22^. Our study focused on investigating the profiles of semi-polar metabolites and SCFAs and BCFAs in bacterial supernatants, but the observed functional effects of the bacterial supernatants may also be mediated by other bacterial-derived compounds including proteins and lipidic components such as ClpB, peptidoglycans, or LPS, previously shown to impact on appetite regulation^51,86,87^. Future work should include other techniques such as proteomics and lipidomics, to comprehensively characterize the complete composition of bacterial supernatants from *B. longum* APC1472 and *L. reuteri* ATCC PTA 6475, including proteins and lipids.

In this study, we used bacterial cell-free supernatants as a model to assess the capability of single bacteria to produce metabolites able to modulate in vitro the expression of genes related to appetite regulation pathways in hypothalamic cells. Currently, investigating gut bacteria-derived metabolites for specific mechanisms using laboratory-grown species in cellular assays is challenging, as the nutrient-rich media can interfere with functional assays and introduce non-specific effects and false positives. Consequently, there is no standardized process to streamline in vitro functional analysis of cell-free bacterial supernatants. Accordingly, we aimed to test an optimized assay media for cell-free supernatants to reduce the masking effect of the nutrient-rich growth media in the in vitro functional assay. We found a differential metabolite profile and specifically, composition of SCFA/BCFA, in CFSs and CCSs supernatants from *B. longum* APC1472 and *L. reuteri* ATCC PTA 6475 (Figs. 1b, 2, 3), which also led to distinct effects of the different supernatants on expression of genes related to appetite regulation pathways in hypothalamic cells (Fig. 4).

The present study used an in vitro model to assess whether bacteria-derived metabolites from *B. longum* APC1472 and *L. reuteri* ATCC PTA 6475 supernatants can impact hypothalamic cells and regulate the expression of genes linked to the central regulation of appetite. A key limitation of the current study lies in the use of an immortalized hypothalamic cell line as an initial in vitro model for screening the effects of the bacterial supernatants on hypothalamic gene expression, which does not recapitulate the cellular heterogeneity, structural organization, or regulatory complexity of the hypothalamus in vivo. The hypothalamus comprises multiple distinct subnuclei, each with specific neuronal and non-neuronal populations that respond to diverse metabolic, hormonal, and neural signals to coordinate homeostatic and non-homeostatic aspects of feeding behavior^17,19^. Our in vitro model lacks this architecture and the dynamic interplay with other central and peripheral systems (e.g., brainstem, vagus nerve, gut, liver, adipose tissue) that critically shape hypothalamic responses. Additionally, by directly applying bacterial supernatants to hypothalamic cells, our model bypasses key physiological barriers, including the intestinal epithelial barrier and BBB, as well as regulatory processes involved in gut-brain communication, such as microbial metabolism, host absorption, and neuroendocrine signaling and overall host system interactions^17,19^. Importantly, microbial-derived metabolites, such as SCFAs^6,88^, trimethylamine-N-oxide (TMAO)^89,90^, among others^91^, have been previously suggested to reach the bloodstream^6,42,88^, cross the BBB and reach the brain where they impact brain function and physiology^6,88^.

We fully acknowledge the limitations of our study and emphasize that the present model serves as a reductionist platform for preliminary in vitro screening of bacterial-derived metabolites with potential neuroactive properties, especially to influence hypothalamic function. This approach enables the identification of candidate strains or compounds for further testing but is not designed to replicate the full complexity of the microbiota-gut-brain interactions. Future studies should incorporate more physiologically relevant models, including in vitro co-culture systems as an approximation of both the gut epithelial and blood-brain barriers, ex vivo experiments with using chambers or brain slice preparations, or in vivo studies to assess whether the observed gene expression changes by bacteria-derived compounds translate into functional effects on neuronal function, behavior, and metabolic regulation.

In the present study, we observed that CCSs have greater differences in the relative abundances of semi-polar metabolites compared to CFSs (Fig. 1b). We hypothesize that CFSs have a higher saturation of the media with other metabolites, potentially with a more variable composition, while these metabolites may increase in relative abundance during incubation in conditioned assay media (CCSs) where growth has ceased. This difference could be also due to microbial metabolism requiring specific metabolites for biosynthesis of others, or because the conditioned environment enhances metabolic activity while reducing metabolite variety and potentially inducing alternative pathways. Even though bacteria have the genetic capability to synthesize certain metabolites, their production depends on specific conditions. Therefore, we suggest that the different metabolite profiles in bacterial supernatants from *B. longum* APC1472 and *L. reuteri* ATCC PTA 6475 might explain the differences between bacterial supernatants and species observed in gene expression in hypothalamic cells. Another limitation of this study is that it relies on in vitro models comparing the effects of the bacterial supernatants produced by individual bacteria in growth media versus conditioned assay media, which are both simplified representations of bacterial metabolite production. In the host, bacteria reside in a highly complex environment, where they interact with a diverse microbial community and compete for nutrients. These microbial interactions can impact metabolic pathways and production in an extent that this model of isolated bacteria does not fully capture. While our study used genomic predictions to infer metabolic capacity and functionally assessed metabolite production in vitro, this approach does not reflect actual pathway regulation under specific culture conditions. Future research should investigate how functional potentials vary in bacterial communities, examine individual and synergistic effects of specific bacteria-derived metabolites, and incorporate transcriptomic profiling under the same conditions to identify actively expressed genes and pathways. Coupling transcriptomic data with metabolomic, lipidomic, and proteomic analyses would provide a more dynamic view of bacterial metabolism, bridging the gap between biosynthetic potential and functional output while identifying microbial pathways most relevant to hypothalamic modulation. Noteworthy, previous work in the group performed comparative genomic studies of *B. longum* strains, including the APC1472, revealing distinct strain-specific genomic features contributing to unique carbohydrate catabolism capabilities^31^. Similar analyses of *L. reuteri* strains, including the ATCC PTA 6475, have shown inter-strain variability in metabolic pathways that may underlie the species-specific functional traits observed in our metabolomic data^92–94^. Overall, these genomic insights support our metabolomic findings and reinforce the importance of both specie- and strain-specific approaches when investigating bacteria-host interactions.

Taken together, our findings demonstrate that the supernatants from *B. longum* APC1472 and *L. reuteri* ATCC PTA 6475 exhibit distinct metabolite profiles, including the SCFAs, consistent with predictions from an in silico analysis of their genomic capability to synthesize or degrade neuroactive compounds. Furthermore, the distinct profile of metabolites, including the SCFAs, present in the bacterial supernatants induced specific changes in the expression of different genes related to appetite regulation in hypothalamic cells. This underscores the specie-specific effects of bacteria-derived metabolites present in bacterial CFSs and CCSs on host expression and function. Understanding the factors in bacterial supernatant production and the impact of their composition and functionality is essential for further elucidating the mechanisms underlying their biological actions and microbe-host interactions. This in vitro study aims to provide foundational insights that could ultimately inform strategies for host’s health, including appetite regulation, through microbiota-targeted interventions.

## Methods

### Determination of the bacterial growth curve

All culturing of *B. longum* APC1472 from the APC Microbiome Ireland and Teagasc Research Center (Cork, Ireland) and *L. reuteri* ATCC PTA 6475 (commercial strain previously named as MM4-1A, acquired from LGC, exclusive distributors for ATCC in Europe) was performed in anaerobic conditions (PLAS-LABS Simplicity 888 Automatic Atmosphere Chamber with anaerobic gas mixture (N_2_ = 80%, H_2_ = 10%, CO_2_ = 10%, temperature 37 °C)).

A glycerol stock from each bacteria was streak-plated individually in De Man, Rogosa, and Sharpe (MRS) medium (Fisher Scientific, BD Difco™ Lactobacilli MRS Broth BD288130) supplemented with 0.05% of L-cysteine hydrochloride (Sigma-Aldrich, C1276-50G) (modified MRS medium, mMRS, from now onwards) until single colonies were visible. In each case, a single colony was then inoculated in liquid mMRS medium and incubated under anaerobic conditions. Growth curves for each bacteria were measured manually, in duplicate, using WPA CO8000 Cell Density Meter and automatically, in triplicate, using Stratus Kinetic Microplate Reader (Cerillo™). Accordingly, a single colony of each bacteria was inoculated in liquid mMRS medium and incubated under anaerobic conditions at 37 °C. The OD600 of the overnight culture was measured on each case. The culture was diluted to OD600 0.05 to add a final volume of 250 µL on each well into a 96-well plate (Sarstedt, Microtest Plate 96 Well 82.1583). After aliquoting all the cultures in triplicate into the plate, the plate was inserted into the plate reader, and the OD600 was monitored for 24 h in a plate shaker (IKA MS 3 Digital Orbital Shaker) in anaerobic conditions at 400 rpm (1 g).

### Preparation of cell-free supernatants and cell-free conditioned supernatants

Cell-free supernatants (CFSs) and cell-free conditioned supernatants (CCSs) from *Bifidobacterium longum* APC1472 and *Limosilactobacillus reuteri* ATCC PTA 6475 were produced (Supplementary Fig. S1).

To produce CFSs and CCSs at early and late stationary phase, streak-plating from a glycerol stock of *B. longum* APC1472 and *L. reuteri* ATCC PTA 6475, respectively, was performed on mMRS agar plates as previously described and incubated until single colonies appeared. A single colony was then inoculated in liquid mMRS medium and incubated until reaching the early stationary phase (*B. longum* APC1472, OD600 of 1.05–1.15 and ~1 × 10^9^ colony-forming unit (CFU)/ml; *L. reuteri* ATCC PTA 6475, OD600 of 1.2–1.3 and ~2.5 × 10^9^ CFU/ml in *L. reuteri* ATCC PTA 6475). Bacterial culture was then centrifuged at 5000 rpm (3968 × g) for 15 min at room temperature. The supernatant of such culture was used as CFSs. The remaining cell pellet was washed twice in the conditioned media, Buffer B (88% of sterile deionized H_2_O, 10% of Hanks Balanced Salt solution - modified, with calcium, with magnesium, without phenol red, liquid, suitable for cell culture (Sigma-Aldrich, 55037 C) and 2% of 4–12 (2-hydroxyethyl)-1-piperazineethanesulfonic acid (HEPES) solution (Sigma-Aldrich, H0887), pH 7.4). Washed bacterial cell pellet was resuspended in the conditioned Buffer B, and the culture was incubated for 4 h, following previous work^29^. The viability of both *B. longum* APC1472 and *L. reuteri* ATCC PTA 6475 during the 4 h incubation was assessed, and no changes were observed during this time (Supplementary Fig. S5). Then each bacterial culture was centrifuged at 5000 rpm (3968 g) for 15 min at 4 ^o^C and CCSs were acquired. Supernatants were harvested at both early stationary phase (OD600 of 1.1–1.2 and ~1.3 × 10^9^ CFU/ml in *B. longum* APC1472 case and OD600 1.3–1.4 and ~2.5 × 10^9^ CFU/ml in *L. reuteri* ATCC PTA 6475 case) and late stationary phase (OD600 of 1.4–1.5 and ~2 × 10^9^ CFU/ml in *B. longum* APC1472 case and OD600 1.55–1.7 and ~3×10^9^ CFU/ml in *L. reuteri* ATCC PTA 6475 case). An aliquot of each culture from each growth phase after the washes was collected to assess cell viability as described below. The pH of the supernatants was adjusted to pH 7.4 with 10 N NaOH, and CFSs and CCSs were filter sterilised through a 0.22 µm filter. The bacterial pellet was used to confirm the purity of the culture via Sanger Sequencing (Eurofins Genomics) of 16S rRNA gene.

This study also sought to compare the metabolite composition of CFSs and CCSs of *B. longum* APC1472 and *L. reuteri* ATCC PTA 6475 to evaluate whether using an optimized assay medium (conditioned non-growth/assay media (Buffer B)) could reduce interference from nutrient-rich growth media (mMRS) and prevent potential masking effects of bacterial cell-free supernatant-derived metabolites in the in vitro assays. Accordingly, CFSs refer to cell-free bacterial supernatants containing metabolites, enzymes, and other molecules secreted by the bacteria during culturing in the growth media (mMRS). CCSs refer to cell-free bacterial supernatants where previously active cells have been moved to a conditioned non-growth/assay media (Buffer B) in order to incubate for a certain period of time (4 h in this study). For in vitro assays, controls consist of the respective media used to prepare the supernatants—mMRS for cell-free supernatants (CFSs) and Buffer B for cell-conditioned supernatants (CCSs)—without bacterial inoculation.

### Cell viability/plate count enumeration of *B. longum* APC1472 and *L. reuteri* ATCC PTA 6475 to produce cell-free supernatants and cell-free supernatants in conditioned media

Cell viability was determined for *B. longum* APC1472 and *L. reuteri* ATCC PTA 6475 and each growth phase (early and late stationary) by counting the colony forming units/mL (CFU/mL, 2 replicates and 3 consecutive dilutions on each case). The same process was performed for the culture in the conditioned media of Buffer B after the two washes (before the incubation time) and every hour within the 4 h incubation in the conditioned media (Supplementary Fig. S5). The purity of the bacterial cultures was also confirmed by Sanger Sequencing (Eurofins Genomics) 16S rRNA gene. OD600 was also monitored in the same time points as aliquots were collected for cell viability assessment.

### Bacterial DNA extraction

Total bacterial DNA was isolated using the QIAamp Fast DNA Stool Mini kit (Qiagen, 51604) following the modified manufacturer’s instructions: after the addition of pre-heated IhibitEX Buffer (95 °C), and zirconia/silica beads (BioSpec), samples were Bead-beaten (FastPrep 24™, MP Biomedicals™, 116004500) twice for 20 s with 1 min incubation period on ice in between. Samples were placed on heat block for 5 min at 95 °C before continuing with manufacturer’s instructions. Quality and quantity of the isolated DNA were assessed using NanoDrop ND1000 spectrophotometer (Thermo Scientific) and kept at −20 °C until further analysis.

### Bacterial amplification of 16S rRNA region

To confirm the purity of all cultures used in the current study for the bacterial supernatants production, the extracted DNA was used as a template for PCR targeting the bacterial 16S ribosomal RNA gene using the universal primers 27F2 and 1492R3 (Supplementary Table S1) (5 μM, Integrated DNA Technologies) and Phusion High-Fidelity PCR Master Mix with HF Buffer (Thermo Fisher Scientific, F531) in a MiniAMP Plus Thermal Cycler (Applied Biosystems).

### Agarose gel electrophoresis

PCR products were analyzed on 2% agarose gel (Invitrogen, UltraPureAgarose 16500500 and Thermo Scientific, 50X TAE Electrophoresis Buffer, B49) and visualized by SYBR Safe DNA Gel Stain (Invitrogen, 533102) staining and loading dye (Invitrogen, Gel Loading Buffer II AM8546G). Electrophoresis/migration was run at 100 V, 400 mA for 30–45 min.

### Clean-up of PCR products

PCR products were purified using the QIAquick PCR Purification Kit (Qiagen, 28106), following the manufacturer’s instructions.

### Sanger Sequencing

The purified PCR products were sequenced by Sanger Sequencing performed by Eurofins Genomics. Obtained sequences were analyzed in BioEdit 7.2.5/Mega 11.0.10 and identified using Basic Local Alignment Search Tool (BLAST) server at the National Center for Biotechnology Information (NCBI), and alignment with the respective source sequence was performed using MultiAlin^95^.

### Whole genome sequencing

For *B. longum* APC1472, whole-metagenome shotgun libraries preparation, indexing, clean-up of the PCR products, sample size and concentration of the samples, and sequencing of the pooled library on the Illumina NovaSeq 2 × 150 bp sequencing with ~500 Mb raw date per sample and SNP/Indel analysis were performed by Genewiz Azenta Life Sciences.

### Genomic analysis, assembly, and annotation

Quality filtering and trimming were performed using the default setting in Trimmomatic version 0.40 and cutadapt version 2.0. Resulting high-quality paired-end reads were assembled into contings using SPAdes version 3.15.4^96^ with default parameters. The assembly was annotated with Prokka version 1.14.6^97^ using default parameters.

The annotated bacterial genomes of the in-house strain *B. longum* APC1472 (NCBI accession number: GCA_002833115.1) and the commercial strain *L. reuteri* ATCC PTA 6475 (accessed in NCBI with accession number: GCA_000159475.2), were further analyzed in Python version 3.10.6 to generate a table of the Kegg Orthologs identified in the genomes was constructed. With that table as an input, the software Omixer-RPM version 1.1^98^ was used with default parameters to identify the presence of specific bacterial pathways previously defined in the literature as gut-brain modules (GBMs)^32^. This approach informs on whether the single bacteria *B. longum* APC1472 and *L. reuteri* ATCC PTA 6475 have partial or total a potential to fulfil the pathway for the synthesis/degradation of related neurobiological compounds.

*L. reuteri* ATCC PTA 6475 was originally isolated from human breast-milk (a Finnish mother, designated MM4-1A then) by researchers at BioGaia (AB, Stockholm, Sweden), and then deposited at the American Type Culture Collection on December 21st, 2004, when received the designation *L. reuteri* ATCC PTA 6475 (GenBank accession number ACGX02000000, sequence ACGX02000001–ACGX02000007; NCBI BioProject Accession PRJNA31511 when sequenced as part of the Human Microbiome Project, GCA_000159475.2)^99^. The genome annotation of this bacteria was previously shown to yield 2019 protein-encoding genes, 71 tRNA, and 18 rRNA genes^94,99,100^ and was shown to belong to the MLSA Clade II as determined by Oh et al. 2010^92,93,99,101^.

### Untargeted semi-polar metabolite analysis

Semi-polar metabolite profiling was performed by MS-OMICS (Vedbaek, Denmark). First, semi-polar metabolite analysis was carried out using a Thermo Scientific Vanquish LC coupled to a Orbitrap Exploris 240 MS (Thermo Scientific). An electrospray ionization interface was used as ionization source. Analysis was performed in positive and negative ionization mode under polarity switching. The UPLC was performed using a slightly modified version of the protocol described by Doneanu et al.^102^. Peak areas were extracted using the Compound Discoverer 3.3 (Thermo Scientific). Identification of compounds were performed at four confidence levels based on an in-house library of molecule standards but only the two highest levels were used for further analyses; Level 1: identification by retention times (compared against in-house authentic standards), accurate mass (with an accepted deviation of 3ppm), and MS/MS spectra (results in main Figs. 1b–3), Level 2a: identification by retention times (compared against in-house authentic standards), accurate mass (with an accepted deviation of 3ppm) (level 2a for metabolites of interest shown in Supplementary Fig. S3). The resulting count table was CLR-transformed to account for the inherent compositionality of multi-omic data^103^ using the R package vegan v2.6.6.1. Overall composition of the samples was assessed using Principal Component Analysis (PCA), performed over Aitchison distance (Euclidian distance over CLR-transformed data), as recommended for compositional data^103^. Similarly, differences between groups were assessed by permutational multivariate analysis of variance (PERMANOVA) using the function adonis2 from the vegan package, with 1e4 permutations. Differential abundance analysis of was performed using the tidymodels approach and the tidyverse package ecosystem v2.0.0^103^. These changes were also assessed using ANOVA followed by Tukey-adjusted post-hoc comparisons test (*p* adjusted value cut-off of 0.05).

In the semi-polar metabolite analysis, a total of 4748 compounds were detected in the samples of CFSs and CCSs from *B. longum* APC1472 and *L. reuteri* ATCC PTA 6475 at early and late stationary phase. From this, 87 metabolites could be identified as level 1, 57 compounds were annotated by accurate mass and matched to the retention time of reference standards run on the same system (level 2a). From the rest, 1023 compounds were annotated by exact mass and isotope pattern and reference count (level 3), and 3496 compounds were distinguished from the background, but they remain unknown. Metabolites identified were classified in chemical classes (Fatty Acids and conjugates, Amino Acids and derivatives, Purines, pyrimidines and derivatives, Lipids and lipid-like molecules, Biotin and derivatives, Carbohydrates and carbohydrate conjugates, and Others) according to the Human Metabolome Database^88^.

### Short- and branched-chain fatty acid profile

Short- and branched-chain fatty acids (SCFAs and BCFAs) targeted metabolomics was performed by MS-OMICS (Vedbaek, Denmark). Accordingly, samples were acidified using hydrochloride acid, and deuterium labelled internal standards were added. All samples were analyzed in a randomized order. Analysis was performed using a high polarity column (Zebron™ ZB-FFAP, GC Cap. Column 30 m x 0.25 mm × 0.25 μm) installed in a GC (7890B, Agilent) coupled with a quadropole detector (5977B, Agilent). The system was controlled by ChemStation (Agilent). Raw data was converted to netCDF format using Chemstation (Agilent), before the data was imported and processed in Matlab R2021b (Mathworks, Inc.) using the PARADISe software described by Johnsen et al. 2017^104^. The count table of SCFA and BCFAs metabolites was analyzed in the same way as described before in the Untargeted semi-polar metabolite analysis.

In the SCFAs and BCFAs analysis, ten compounds were analyzed (acetate, formate, propionate, 2-methylpropanoic acid, butyrate, 3-methylbutanoic acid, pentanoic acid, 4-methylpentatoic acid, hexanoic acid, and heptanoic acid).

### Cell culture

Embryonic (mHypoE-N41, CLU121) and adult (mHypoA2/28, CLU188) mouse hypothalamic immortalized cell lines (purchased from CELLutions Biosystems from CEDARLANE® (Ltd., Ontario, Canada), distributed by Tebu-bio (Inc., Peterborough)) were grown and maintained in 1x DMEM medium (Sigma-Aldrich, D5796), supplemented with 10% heat-inactivated FBS (Sigma-Aldrich, F7524), 1% Non-Essential Amino Acids Solution (NEAAs) (Gibco™MEM, 11140-050) and 1% penicillin/streptomycin (Sigma-Aldrich, P4333)). Cells were incubated at 37 °C and 5% CO_2_ in a humidified atmosphere and grown in monolayer culture.

### Analysis of mycoplasma contamination in mHypoA2/28 and mHypoE-N41 cell lines

The presence of mycoplasma contamination was discarded in mHypoE-N41 and mHypoA2/28 cell cultures, using the MycoAlert™ PLUS Mycoplasma Detection Kit (Lonza, LT07-518).

### Cell viability test via 3‑(4, 5‑Dimethylthiazolyl2)‑2,5‑Diphenyltetrazolium Bromide (MTT) Assay

mHypoE-N41 and mHypoA2/28 cells (0.02 × 10^6^ and 0.015 × 10^6^, respectively per well) were seeded in 96-well plate (Sarstedt, 82.1583), in 200 μL of complete growth medium (1x DMEM medium, supplemented with 10% heat-activated FBS, 1% NEAAs and 1% penicillin/streptomycin and incubated at 37 °C and 5% CO_2_ in a humidified atmosphere. When cells reached confluency, cells were incubated in DMEM media free from FBS, NEAA and antibiotics for 1 h. Then, cells were acutely exposed (2 h) to CFSs or bacterial cell-free supernatants in conditioned media (Buffer B) of *B. longum* APC1472 and *L. reuteri* ATCC PTA 6475 at different concentrations (dilution 1/3, 1/5, 1/10, 1/20 of CFSs and 1/2, 1/3, 1/5, 1/10 of the supernatants in the conditioned media Buffer B, diluted in DMEM media). After the acute exposure, treatments were replaced by fresh DMEM media free from FBS, NEAA, and antibiotics and mHypoE-N41 and mHypoA2/28 cells were incubated with 0.5 mg/mL 3-(4,5-dimethylthiazol-2-yl)-2,5-diphenyltetrazolium bromide (MTT) (Sigma-Aldrich, M2128) for 3 h at 37 °C. MTT solution was by DMSO (Sigma-Aldrich, D8418), and absorbance was measured at 590 nm using the Biotek microtiter plate reader (Synergy HT), and results were expressed as percentage cell viability compared to untreated cells (only with DMEM media, negative control). Triton-X100 (1% (v/v) was added as an additional variable in every MTT assay as positive control for cell death.

### Acute exposure to cell free-supernatants and cell-free conditioned supernatants from *B. longum* APC1472 and *L. reuteri* ATCC PTA 6475 in hypothalamic mouse cell lines

mHypoE-N41 and mHypoA2/28 cells (0.15 × 10^6^ and 0.25 × 10^6^, respectively on each well) with complete growth medium (DMEM), supplemented with heat-inactivated FBS, 1% Penicillin/Streptomycin, and 1% NEAAs) were seeded in a pre-coated with 50 μg/mL poly-L-Lysine (Sigma-Aldrich, P4707) 24-well plates (Sarstedt, 83.3920.300). Cells were incubated for 48 h at 37 °C and 5% CO_2_. Then, media was replaced with DMEM media free from FBS, NEAA and antibiotics and cells were incubated for 1 h. Cells were incubated for 2 h (acute exposure) to CFSs and bacterial supernatants in conditioned media (Buffer B) of *B. longum* APC1472 and *L. reuteri* ATCC PTA 6475 (dilution 1/10 for CFSs and 1/3 for bacterial supernatants in conditioned media (CCSs), in DMEM free from FBS, NEAA and antibiotics). After the acute exposure, cells were lysed and stored at −80 °C for future RNA extraction.

### Cellular RNA isolation, reverse-transcription and quantitative real-time PCR

Cellular total RNA was extracted using the High Pure RNA Isolation Kit (Roche, 12033674001) according to the manufacturer’s recommendations. Equal amounts of RNA were first reverse transcribed to cDNA using High-Capacity cDNA Reverse Transcription Kit (Applied Biosystems, 4368814). Following reverse transcription, real-time quantitative Polymerase Chain Reaction (PCR) was performed using qPCRBIO SyGreen Mix Lo-ROX (PC Bio, PB20.11-51). mRNA expression levels of respective genes (Supplementary Table S1) were measured using a LightCycler 480 II thermo cycler (Roche). Target and housekeeping (β-actin or *Actb*) genes were amplified with probes designed by Integrated DNA Technologies (Supplementary Table S1). Each sample was analyzed in triplicate for both target gene and reference gene, and the relative mRNA expressions were calculated using the 2^-ΔΔCt^ method (cycle threshold (Ct)^105^. Target genes (*Ghrl*, *Ghsr*, *Glp1r* and *Npy1r*) were selected based on their established relevance to appetite regulation^14,20,21,46^ and potential interplay with the microbiota-gut-brain axis^15,29,42,47–49^, and their baseline expression levels in the in vitro cell model used in this study (Supplementary Table S4).

### Statistical analyses

Metabolomic data and plotting were analyzed and performed using R Studio (2023.12.1). Sample dissimilarity was assessed using Aitchison distance (Euclidean distance over CLR-transformed data)^103^, and permutational multivariate analysis of variance (PERMANOVA). Since no differences were observed in dissimilarity in supernatants between early and late stationary phase (growth phase as a factor), differential abundance analysis was calculated for each metabolite in the final CLR-transformed concentration, using the formula: *metabolite ~ bacterial species + media*. These changes were assessed using ANOVA followed by Tukey-adjusted post-hoc comparisons (*p* adjusted value cut-off of 0.05). Further detail of the statistical analyses of metabolomic data can be found on https://github.com/Benjamin-Valderrama/cuesta2025_probiotic_appetite/tree/main/outputs.

Statistical analysis of gene expression and cell viability (MTT assay) was conducted in SPSS (IBM, SPSS Statistics 28.0.1.1). To align with the statistical analysis of the metabolomics data and since preliminary analysis showed no significant differences in metabolite composition between early and late stationary phases (growth phase as a factor), data were analyzed using two-way ANOVA (media/supernatant type and bacterial species as the two factors) followed by Dunnett’s contrasts for targeted comparisons between *B. longum* APC1472 and *L. reuteri* ATCC PTA 6475 against the reference group (control) for each media/supernatant type. Data are shown as mean ± standard error of the mean (SEM). Statistical significance was set at *p* < 0.05.

## Supplementary information

Document S1.

reporting-summary-flat.

## Data Availability

Metabolomics, GBMs coverage data, and in vitro findings of the present study are publicly available and can be found on https://github.com/Benjamin-Valderrama/cuesta2025_probiotic_appetite/tree/main/data.

## Code availability

The scripts used for processing and analyzing the metabolomic datasets can be found on https://github.com/Benjamin-Valderrama/cuesta2025_probiotic_appetite/blob/main/scripts.
