# Supplementary material for: In vitro assessment of bacterial supernatants on hypothalamic gene expression: implications for appetite regulation
Source: NPJ Biofilms Microbiomes. 2025 Oct 3;11:192. doi: 10.1038/s41522-025-00820-9 (PMC12494950; doi:10.1038/s41522-025-00820-9)
Supplement: Supplementary file 1 — Document S1. [file 41522_2025_820_MOESM1_ESM.docx]

## Supplementary information

*In vitro* assessment of bacterial supernatants on hypothalamic gene expression: implications for appetite regulation

Cristina Cuesta-Marti^*1,2^, Benjamin Valderrama^1,2^, Thomaz Bastiaanssen^1,2^, John F. Cryan^1,2^, Catherine Stanton^2,3^, Siobhain M. O’Mahony^1,2^, Gerard Clarke^2,4^, Harriët Schellekens^*1,2^

1 Department of Anatomy and Neuroscience, University College Cork, Cork, Ireland

2 APC Microbiome Ireland, University College Cork, Cork, Ireland

3 Teagasc Food Research Centre, Moorepark, Fermoy, Co. Cork, Ireland

4 Department of Psychiatry and Neurobehavioural Science, University College Cork, Cork, Ireland

*Corresponding authors: h.schellekens@ucc.ie and CCuestaMarti@ucc.ie

### Supplementary Figure S1.


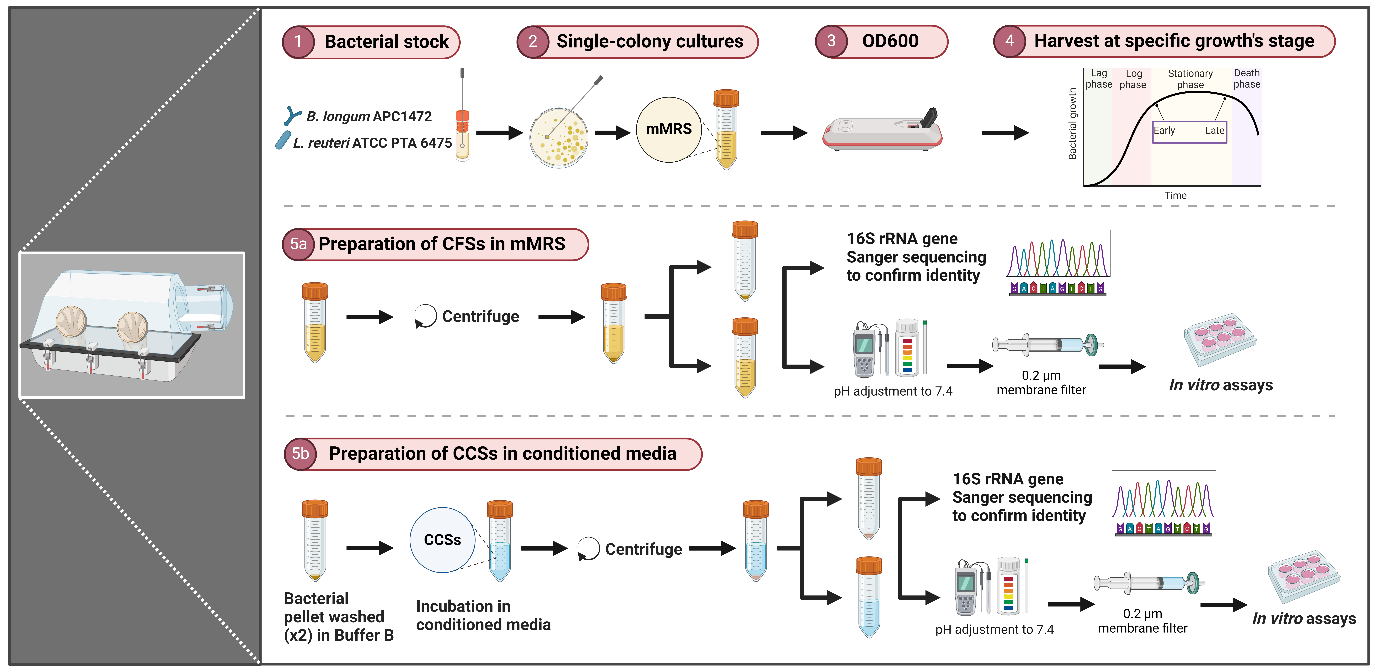


Overview of the methodology for production of bacterial cell-free supernatants (CFSs) and cell-free conditioned supernatants (CCSs) from *Bifidobacterium longum* APC1472 and *Limosilactobacillus reuteri* ATCC PTA 6475. mMRS, modified De Man, Rogosa and Sharpe (MRS) medium; OD, optical density; rRNA, ribosomal RNA gene.

### Supplementary Figure S2.

*
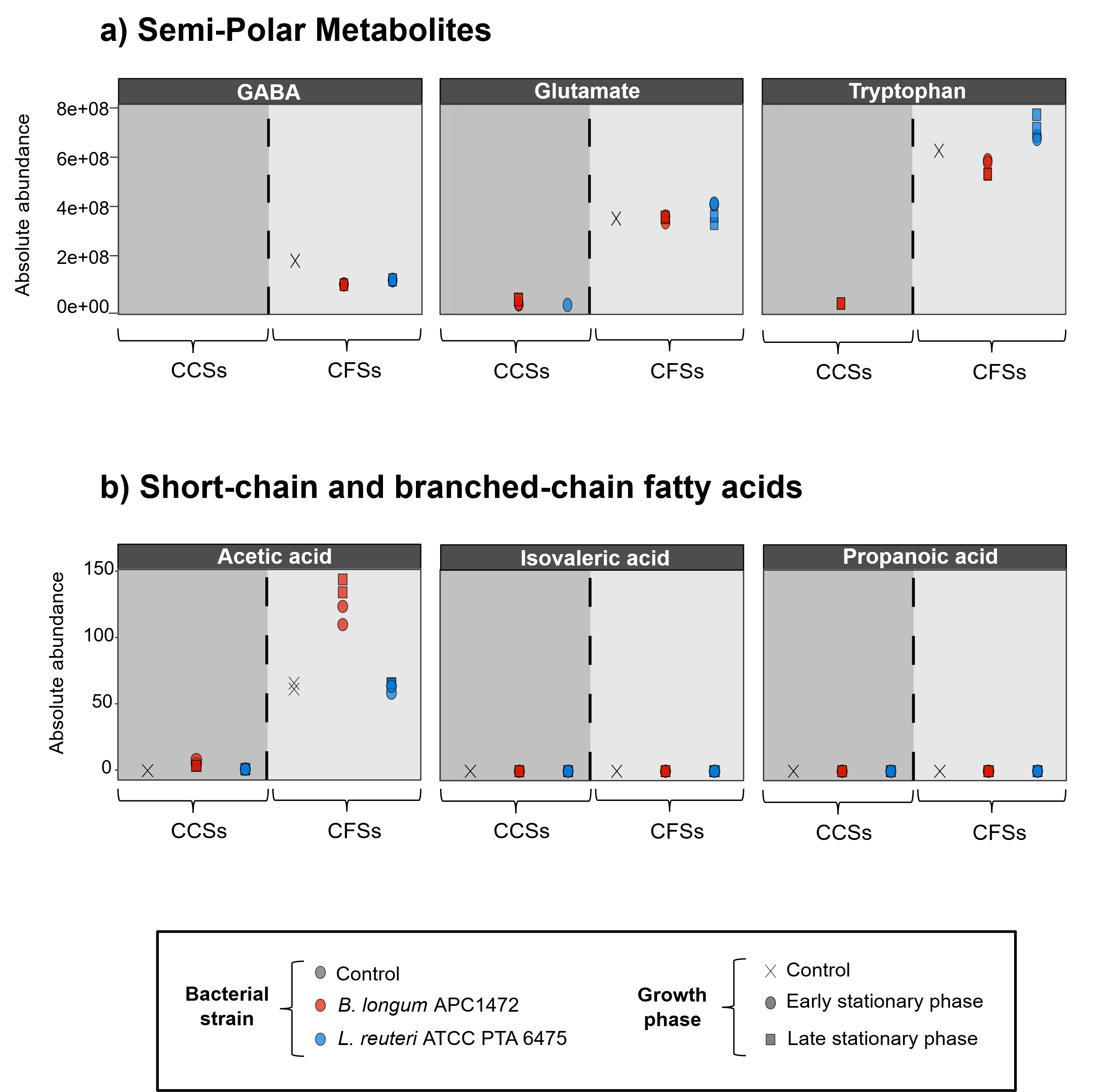
*

Raw abundance of of (a) semi-polar metabolites and (b) acetate, propionate and isovaleric acid in CFSs or CCSs from *B. longum* APC1472 and *L. reuteri* ATCC PTA 6475 when grown in mMRS or Buffer B medias, respectively, compared to control (N = 4 group/media). Further detail of the statistical analyses of metabolomic data can be found on <https://github.com/Benjamin-Valderrama/cuesta2025_probiotic_appetite/tree/main/outputs>.

### Supplementary Figure S3.


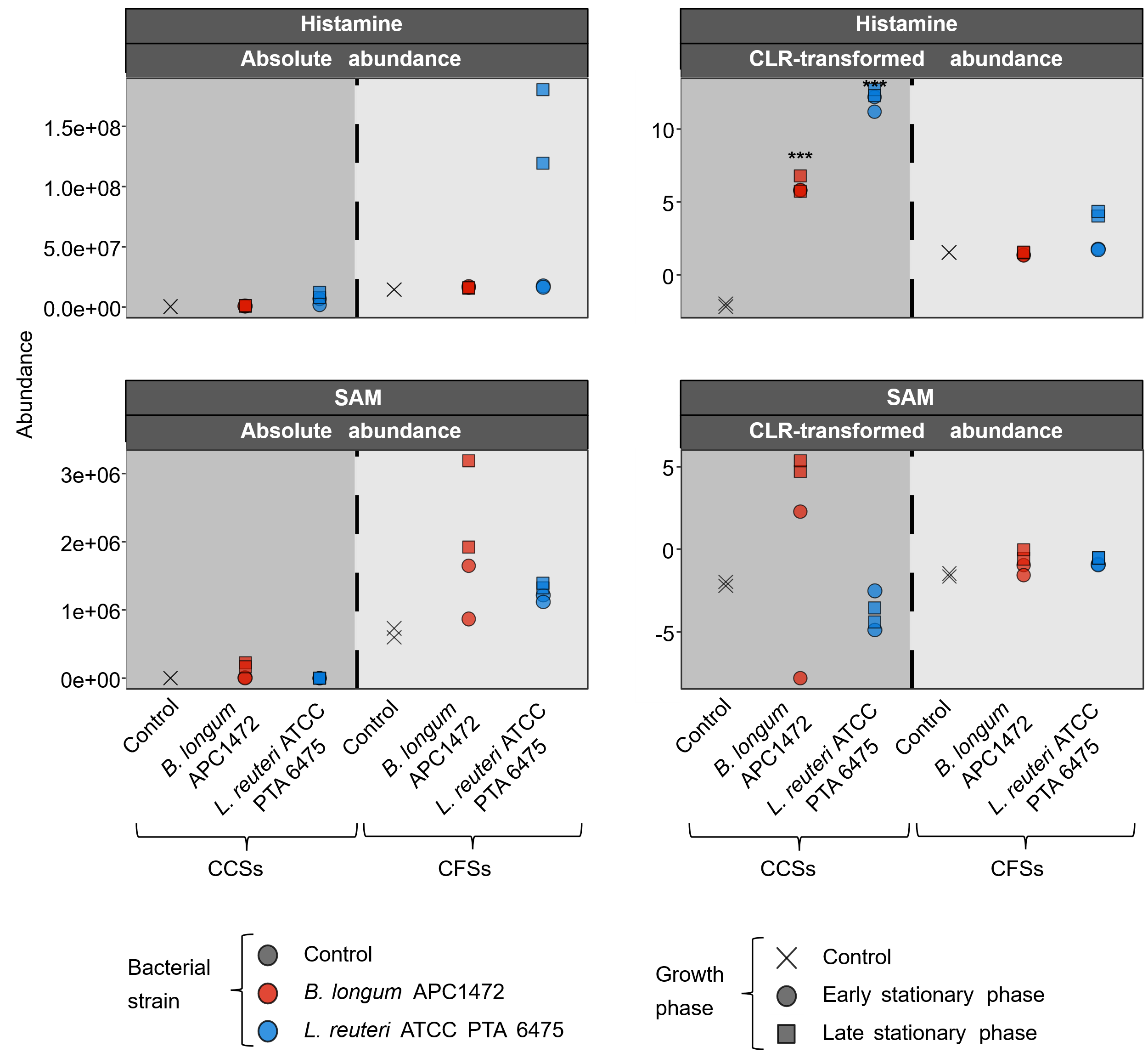
 Raw abundance and centered log-ratio (CLR)-transformed abundance of the semi-polar metabolites histamine and S-adenosyilmethionine (SAM) in CFSs and CCSs from *B. longum* APC1472 and *L. reuteri* ATCC PTA 6475 when grown in mMRS or Buffer B medias, respectively, compared to control (N = 4 group/media). Significant differences assessed by ANOVA followed by Tukey-adjusted post-hoc comparisons (metabolite ~ bacterial specie + media) (p = p.adjusted value, p* < 0.05, **p < 0.01, ***p < 0.001). Further detail of the statistical analyses of metabolomic data can be found on https://github.com/Benjamin-Valderrama/cuesta2025_probiotic_appetite/tree/main/outputs.

### Supplementary Figure S4.


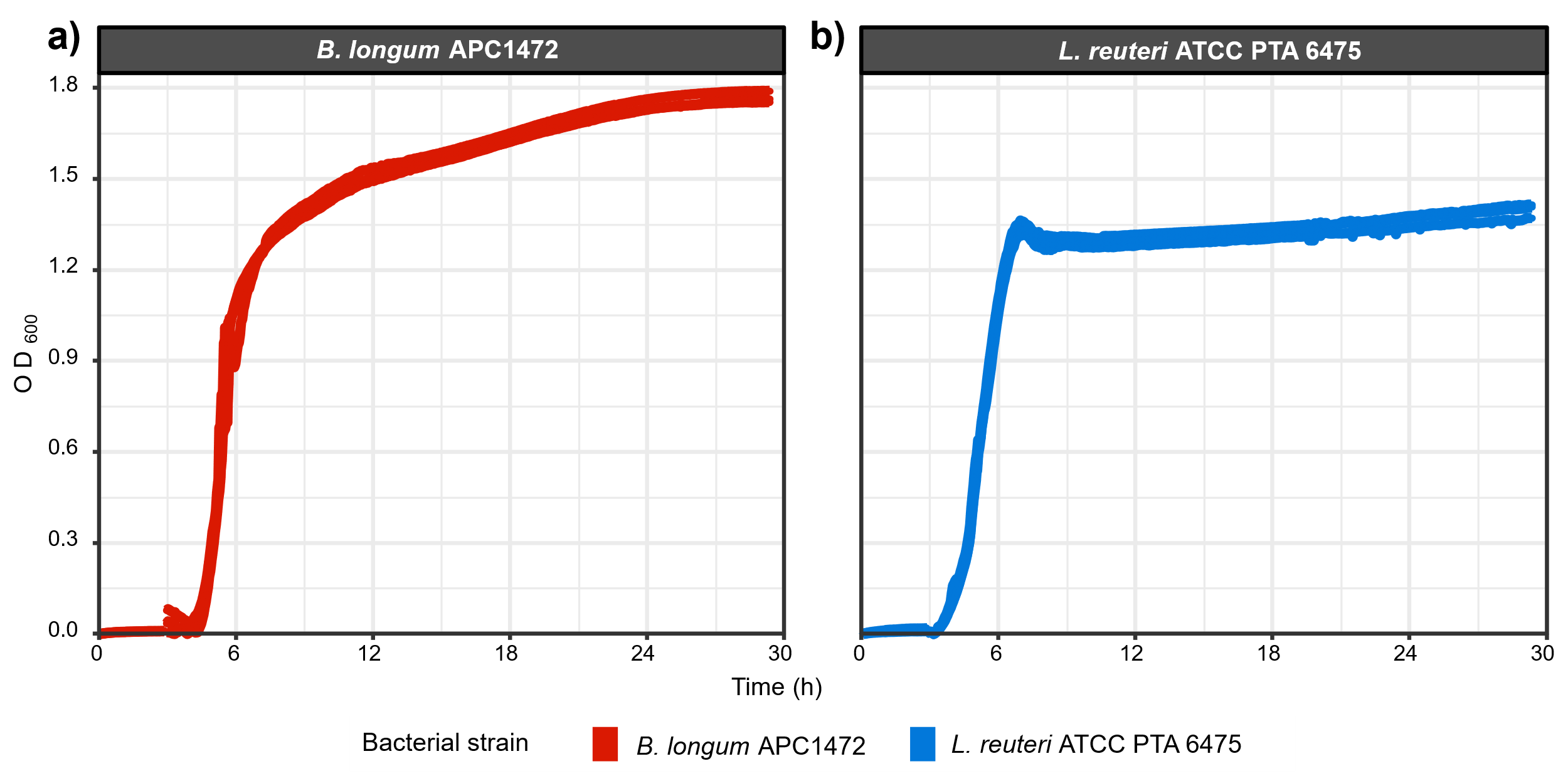
 Growth curve (OD600) *B. longum* APC1472 (a) and *L. reuteri* ATCC PTA 6475 (b) in mMRS media throughout the time in hours (h). N = 3 biological replicates. OD600: optical density at 600 nm of wavelength, mMRS: modified De Man–Rogosa–Sharpe.

### Supplementary Figure S5.

Viable cell count (cfu/ml) of *B. longum* APC1472 and *L. reuteri* ATCC PTA 6475 during the 4 hour (h) incubation in conditioned media (Buffer B) after been grown in modified De Man–Rogosa–Sharpe (mMRS). Data represented as one batch per bacterial strain checking cell viability on three dilution factor on each case. CFU, colony-forming units.

### Supplementary Figure S6.


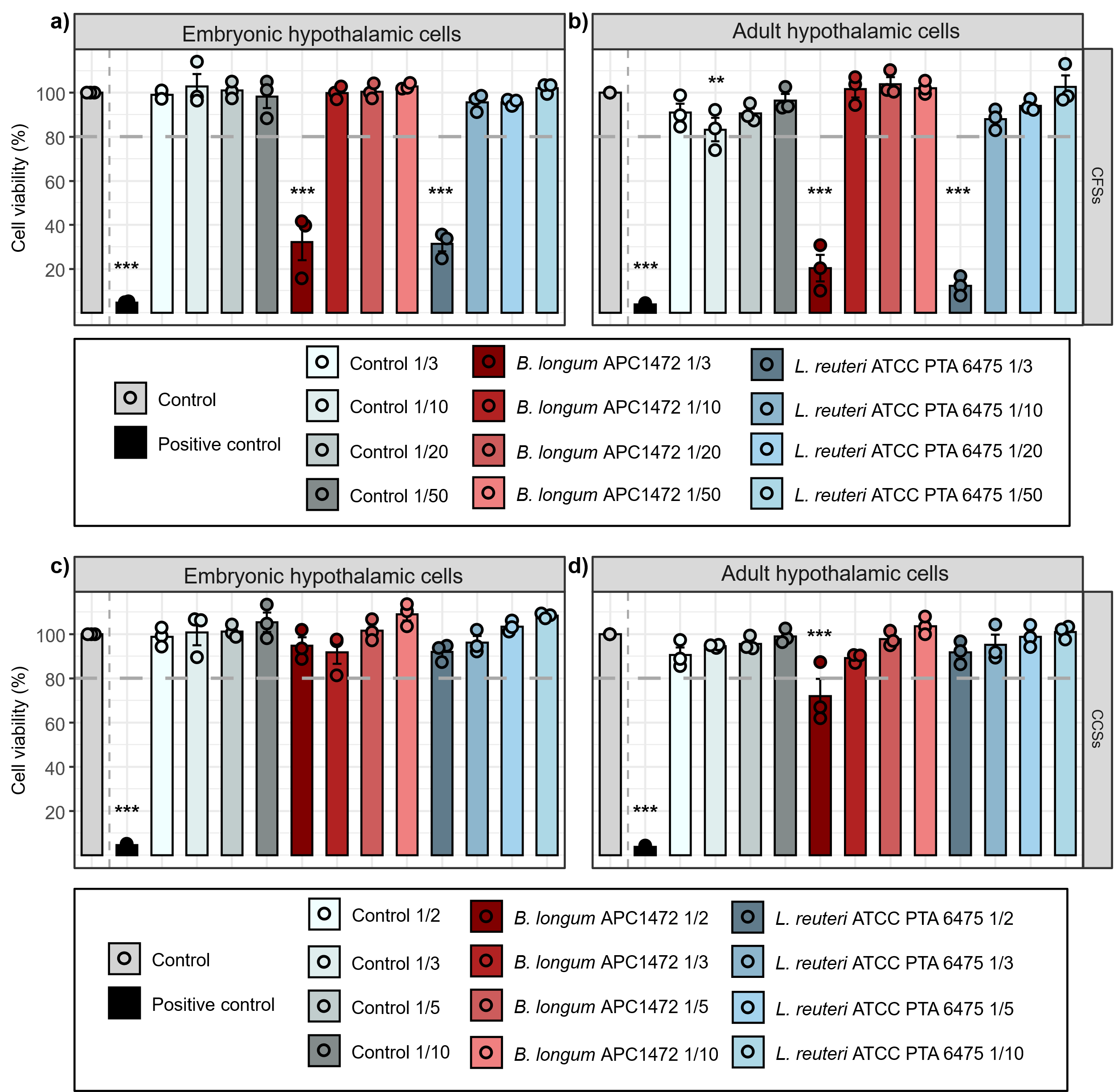
 Cell viability of hypothalamic embryonic mHypoE-N41 (a, c) and adult mHypoA2/28 cells (b, d) after incubation with different dilutions of (a, b) cell-free supernatants (CFSs) and (c, d) cell-free conditioned supernatants (CCSs) from *B. longum* APC1472 and *L. reuteri* ATCC PTA 6475 . a-d) N = 3 group/media/dilution factor. Data are shown as mean ± SEM. Cell viability calculated as percentage cell viability compared to untreated cells (only with DMEM media, positive control). Data are significantly different (*p < 0.05, **p < 0.01, ***p < 0.001) according to two-way ANOVA followed by Dunnett’s contrasts ((bacteria in the different dilutions) and media as factors).

### Supplementary Table S1.

List of primers with the corresponding sequences for forward and reverse probes (5’-3’) per gene.

| **Gene** | **Primer** | **Primer sequence** |
| --- | --- | --- |
| ***Actb*** | Forward | CTA AGG CCA ACC GTG AAA AG |
|  | Reverse | ACC AGA GGC ATA CAG GGA CA |
| ***Ghrl*** | Forward | GAA TCC AAG AAG CCA CCA G |
|  | Reverse | ATG CCA ACA TCG AAG GGA G |
| ***Ghsr*** | Forward | CTC AAT AAG CAA GCA AGC AAA C |
|  | Reverse | ACC TCA ACA TCA TAA CCA CAA C |
| ***GLP-1R*** | Forward | CAG TGG GGT ACG CAC TTT CT |
|  | Reverse | TAA CGA ACA GCA GCG GAA CT |
| ***Npy1r*** | Forward | CCC ATC TGA CTC TCA CAG GC |
|  | Reverse | AGA GTG GGC CGA AAT ACT GC |
| ***27F2*** | Forward | AGA GTT TGA TYM TGG CTC |
| ***1492R3*** | Reverse | GGN TAC CTT GTT AYG ACT T |

### Supplementary Table S2.

Summary table with statistical data of cell viability of hypothalamic cells after exposure to bacterial supernatants of *B. longum* APC1472 and *L. reuteri* ATCC PTA 6475.

| Embryonic | | | |
| --- | --- | --- | --- |
|  |  | **q (for multiple comparisons),  F (DFn, DFd)** | **P value** |
| Main effect | **Media** | F (1, 56) = 68.8 | <0.001 |
|  | **Bacteria & dilution** | F (13, 56) = 147 | <0.001 |
|  | **Interaction** | F (13, 56) = 23.4 | <0.001 |
| Post-hoc, multiple comparisons | **Control vs. + control** | 20.7, F (13, 56) | <0.001 |
|  | **Control vs. mMRS 1/3** | 0.21, F (13, 56) | >0.999 |
|  | **Control vs. mMRS 1/10** | 0.613, F (13, 56) | >0.999 |
|  | **Control vs. mMRS 1/20** | 0.219, F (13, 56) | >0.999 |
|  | **Control vs. mMRS 1/50** | 0.392, F (13, 56) | >0.999 |
|  | **Control vs. CFSs_*B. longum* APC1472 1/3** | 14.7, F (13, 56) | <0.001 |
|  | **Control vs. CFSs_*B. longum* APC1472 1/10** | 0.012, F (13, 56) | >0.999 |
|  | **Control vs. CFSs_*B. longum* APC1472 1/20** | 0.12, F (13, 56) | >0.999 |
|  | **Control vs. CFSs_*B. longum* APC1472 1/50** | 0.617, F (13, 56) | >0.999 |
|  | **Control vs. CFSs_*L. reuteri* ATCC PTA 6475 1/3** | 15, F (13, 56) | <0.001 |
|  | **Control vs. CFSs_*L. reuteri* ATCC PTA 6475 1/10** | 0.949, F (13, 56) | 0.969 |
|  | **Control vs. CFSs_*L. reuteri* ATCC PTA 6475 1/20** | 0.924, F (13, 56) | 0.975 |
|  | **Control vs. CFSs_*L. reuteri* ATCC PTA 6475 1/50** | 0.45, F (13, 56) | >0.999 |
|  | **Control vs. - control** | 20.7, F (13, 56) | <0.001 |
|  | **Control vs. Buffer B 1/3** | 0.25, F (13, 56) | >0.999 |
|  | **Control vs. Buffer B 1/10** | 0.179, F (13, 56) | >0.999 |
|  | **Control vs. Buffer B 1/20** | 0.271, F (13, 56) | >0.999 |
|  | **Control vs. Buffer B 1/50** | 1.18, F (13, 56) | 0.882 |
|  | **Control vs. CCSs_*B. longum* APC1472 1/3** | 1.13, F (13, 56) | 0.907 |
|  | **Control vs. CCSs_*B. longum* APC1472 1/10** | 1.77, F (13, 56) | 0.47 |
|  | **Control vs. CCSs_*B. longum* APC1472 1/20** | 0.367, F (13, 56) | >0.999 |
|  | **Control vs. CCSs_*B. longum* APC1472 1/50** | 1.99, F (13, 56) | 0.33 |
|  | **Control vs. CCSs_*L. reuteri* ATCC PTA 6475 1/3** | 1.74, F (13, 56) | 0.493 |
|  | **Control vs. CCSs_*L. reuteri* ATCC PTA 6475 1/10** | 0.819, F (13, 56) | 0.989 |
|  | **Control vs. CCSs_*L. reuteri* ATCC PTA 6475 1/20** | 0.763, F (13, 56) | 0.991 |
|  | **Control vs. CCSs_*L. reuteri* ATCC PTA 6475 1/50** | 1.83, F (13, 56) | 0.435 |
| Adult | | | |
|  |  | **q (for multiple comparisons),  F (DFn, DFd)** | **P value** |
| Main effect | **Media** | F (1, 56) = 68.4 | <0.001 |
|  | **Bacteria & dilution** | F (13, 56) = 155 | <0.001 |
|  | **Interaction** | F (13, 56) = 28.7 | <0.001 |
| Post-hoc, multiple comparisons | **Control vs. + control** | 20.8, F (13, 56) | <0.001 |
|  | **Control vs. mMRS 1/3** | 1.94, F (13, 56) | 0.362 |
|  | **Control vs. mMRS 1/10** | 3.61, F (13, 56) | 0.007 |
|  | **Control vs. mMRS 1/20** | 2.03, F (13, 56) | 0.309 |
|  | **Control vs. mMRS 1/50** | 0.754, F (13, 56) | 0.992 |
|  | **Control vs. CFSs_*B. longum* APC1472 1/3** | 17.2, F (13, 56) | <0.001 |
|  | **Control vs. CFSs_*B. longum* APC1472 1/10** | 0.38, F (13, 56) | >0.999 |
|  | **Control vs. CFSs_*B. longum* APC1472 1/20** | 0.852, F (13, 56) | 0.987 |
|  | **Control vs. CFSs_*B. longum* APC1472 1/50** | 0.472, F (13, 56) | >0.999 |
|  | **Control vs. CFSs_*L. reuteri* ATCC PTA 6475 1/3** | 19, F (13, 56) | <0.001 |
|  | **Control vs. CFSs_*L. reuteri* ATCC PTA 6475 1/10** | 2.59, F (13, 56) | 0.101 |
|  | **Control vs. CFSs_*L. reuteri* ATCC PTA 6475 1/20** | 1.26, F (13, 56) | 0.836 |
|  | **Control vs. CFSs_*L. reuteri* ATCC PTA 6475 1/50** | 0.587, F (13, 56) | >0.999 |
|  | **Control vs. - control** | 20.8, F (13, 56) | <0.001 |
|  | **Control vs. Buffer B 1/3** | 2.03, F (13, 56) | 0.308 |
|  | **Control vs. Buffer B 1/10** | 1.15, F (13, 56) | 0.897 |
|  | **Control vs. Buffer B 1/20** | 0.924, F (13, 56) | 0.975 |
|  | **Control vs. Buffer B 1/50** | 0.217, F (13, 56) | >0.999 |
|  | **Control vs. CCSs_*B. longum* APC1472 1/3** | 6.03, F (13, 56) | <0.001 |
|  | **Control vs. CCSs_*B. longum* APC1472 1/10** | 2.31, F (13, 56) | 0.184 |
|  | **Control vs. CCSs_*B. longum* APC1472 1/20** | 0.474, F (13, 56) | >0.999 |
|  | **Control vs. CCSs_*B. longum* APC1472 1/50** | 0.814, F (13, 56) | 0.99 |
|  | **Control vs. CCSs_*L. reuteri* ATCC PTA 6475 1/3** | 1.77, F (13, 56) | 0.476 |
|  | **Control vs. CCSs_*L. reuteri* ATCC PTA 6475 1/10** | 1.04, F (13, 56) | 0.943 |
|  | **Control vs. CCSs_*L. reuteri* ATCC PTA 6475 1/20** | 0.232, F (13, 56) | >0.999 |
|  | **Control vs. CCSs_*L. reuteri* ATCC PTA 6475 1/50** | 0.22, F (13, 56) | >0.999 |

### Supplementary Table S3.

Summary table with statistical data of modulation of hypothalamic expression of genes involved in appetite regulation by supernatants of *B. longum* APC1472 and *L. reuteri* ATCC PTA 6475. Table shows the q (for multiple comparisons), F (DFn, DFd) and p values).

|  | Embryonic | | | | | | | | |
| --- | --- | --- | --- | --- | --- | --- | --- | --- | --- |
|  | **Gene** | **Ghrl** | | **Ghsr** | | **Glp1r** | | **Npy1r** | |
| Main effect | **Interaction** | F (2, 34) = 0.0596 | 0.942 | F (2, 34) = 0.497 | 0.613 | F (2, 34) = 1.16 | 0.326 | F (2, 34) = 2.20 | P=.127 |
|  | **Media** | F (1, 34) = 0.0123 | 0.912 | F (1, 34) = 2.16 | 0.151 | F (1, 34) = 10.1 | 0.003 | F (1, 34) = 5.51 | P=.025 |
|  | **Treatment** | F (2, 34) = 0.845 | 0.438 | F (2, 34) = 9.11 | <0.001 | F (2, 34) = 0.789 | 0.463 | F (2, 34) = 1.10 | P=.345 |
| Post-hoc, multiple comparisons | **CFSs_Control vs. *B. longum* APC1472** | -0.046, q = 0.16 | 0.979 | -0.668, q = 1.69 | 0.163 | -0.051, q = 0.266 | 0.942 | 0.833, q = 0.47 | 0.834 |
|  | **CFSs_Control vs. *L. reuteri* ATCC PTA 6475** | -0.205, q = 0.716 | 0.67 | -1.29, q = 3.27 | 0.005 | -0.132, q = 0.685 | 0.692 | -0.143, q = 0.792 | 0.618 |
|  | **CCSs_Control vs. *B. longum* APC1472** | -0.185, q = 0.648 | 0.717 | -0.111, q = 0.279 | 0.937 | 0.351, q = 1.83 | 0.126 | 0.290, q = 1.61 | 0.187 |
|  | **CCSs_Control vs. *L. reuteri* ATCC PTA 6475** | -0.301, q = 1.05 | 0.448 | -0.909, q = 2.3 | 0.048 | 0.210, q = 1.09 | 0.425 | 0.361, q = 2.01 | 0.088 |
|  | **Adult** | | | | | | | | |
|  | **Gene** | **Ghrl** | | **Ghsr** | | **Glp1r** | | **Npy1r** | |
| Main effect | **Interaction** | F (2, 34) = 1.67 | 0.202 | F (2, 34) = 9.05 | <0.001 | F (2, 34) = 5.71 | 0.007 | F (2, 34) = 2.89 | 0.069 |
|  | **Media** | F (1, 34) = 0.0280 | 0.868 | F (1, 34) = 44.4 | <0.001 | F (1, 34) = 15.8 | <0.001 | F (1, 34) = 3.12 | 0.086 |
|  | **Treatment** | F (2, 34) = 0.281 | 0.757 | F (2, 34) = 14.1 | <0.001 | F (2, 34) = 3.34 | 0.047 | F (2, 34) = 2.78 | 0.076 |
| Post-hoc, multiple comparisons | **CFSs_Control vs. *B. longum* APC1472** | 0.265, q = 0.845 | 0.582 | -0.537, q = 0.605 | 0.747 | -1.21, q = 3.36 | 0.004 | -0.335, q = 1.75 | 0.145 |
|  | **CFSs_Control vs. *L. reuteri* ATCC PTA 6475** | -0.084, q = 0.266 | 0.942 | -0.885, q = 0.997 | 0.482 | -1.18, q = 3.27 | 0.005 | -0.35, q = 1.83 | 0.126 |
|  | **CCSs_Control vs. *B. longum* APC1472** | 0.056, q = 0.18 | 0.973 | -5.86, q = 6.6 | <0.001 | 0.507, q = 1.41 | 0.264 | 0.244, q = 1.28 | 0.324 |
|  | **CCSs_Control vs. *L. reuteri* ATCC PTA 6475** | 0.362, q = 1.15 | 0.389 | -4.8, q = 5.41 | <0.001 | -0.121, q = 0.336 | 0.91 | -0.182, q = 0.95 | 0.512 |

### Supplementary Table S4.

Manufacturer information with marker expression of the embryonic hypothalamic mHypoE-N41 (CLU121) and adult hypothalamic mHypoA2/28 (CLU188) mouse cell lines from manufacturer (CEDARLANE®). Blank space in expression column refers to marker whose expression has not been analysed. +: expressed marker, -: non-expressed marker, strong/weak/very weak: levels of expression, N/A: non-applicable,

| **Marker** | **Expression in mHypoE-N41 (CLU121)** | **Marker** | **Expression in mHypoA2/28 (CLU188)** |
| --- | --- | --- | --- |
| **5-HT7 receptor** |  | 5-HT2C receptor | very weak |
| **Adiponectin receptor 1** | + | Agouti-Related Peptide (AgRP) | very weak |
| **Adiponectin receptor 2** | + | Androgen Receptor (AR) | + |
| **Adiponutrin (cDNA)** | + | Arginine vasopressin receptor 1a (AVPR1a) | + |
| **Adiponutrin (one-step)** | - | Arginine-vasopressin (AVP) | weak |
| **Agrp** | + | BDNF | N/A |
| **AhR** | + | Ciliary Neurotrophic Factor Receptor (CNTFR) | + |
| **Angiotensinogen** | strong | Corticotropin Releasing Hormone (CRH) | - |
| **Angiotension Receptor 1** | strong | Estrogen Receptor alpha (ER alpha) | + |
| **Angiotension Receptor 2** | - | Estrogen Receptor beta (ER beta) | + |
| **AR** | + | GABAa receptor | N/A |
| **Arnt2** | + | GABAb receptor | N/A |
| **Aromatase** | + | Galanin | + |
| **Avp** | - | Ghrelin (Gh) | strong |
| **AVPR1a** | + | Ghrelin Receptor (GHSR) | + |
| **AVPR1b** | + | Glucagon-like Peptide Receptor 1 (Glp1R) | + |
| **BDNF** | + | Glucagon-like Peptide Receptor 2 (Glp2R) | - |
| **BRS-3** |  | Glucocorticoid receptor (GR) | N/A |
| **Calcium Receptor (CaR)** | + | GnRH | + |
| **CART** | ? | Gpr 54 (Kiss-1 Receptor) | + |
| **CB1 receptor** | + | Huntingtin-associated protein 1 (HAP1) | + |
| **cc1αb** |  | Insulin Receptor (IR) | + |
| **cc1αc** |  | Leptin Receptor (ObRb) | + |
| **ChAT** | - | Melanocortin Receptor (MC3R) | weak |
| **chemerin** | + | Melanocortin Receptor (MC4R) | + |
| **chemerinR** | + | Neurokinin B (TAC2) | - |
| **CNTFR** | + | Neuropeptide Y (NPY) | - |
| **CRFR1** | + | Neurotensin (NT) | - |
| **CRFR2** | + | NPY Y1R | + |
| **CRH(idt)** | weak | NPY Y5R | + |
| **CRLR** | + | Orexin | - |
| **DAT** | + | Orexin R1 | strong |
| **Dpp4** | - | Orexin R2 | + |
| **Dynorphin** | - | Oxytocin Receptor | + |
| **ER α** | + | Proglucagon (ProGlu) | strong |
| **ER β** | + | Proopiomelanocortin (POMC) |  |
| **FIAF** | + | Sim1 | - |
| **Gad 1 (Gad 67)** | + | Steroidogenic Factor-1 (SF-1) | + |
| **Gad 2 (Gad 65)** | + | Tyrosine Hydroxylase (TH) | weak |
| **Gal** | + | Urocortin | strong |
| **GALP** | + |  |  |
| **GCK** | + |  |  |
| **GFAP** |  |  |  |
| **Ghrelin** | + |  |  |
| **Ghrelin variant** | + |  |  |
| **GHRH** *(southern)* | + |  |  |
| **GHSR1a** | strong |  |  |
| **Glp1R** | + |  |  |
| **Glp2R** | weak |  |  |
| **Glucagon receptor** | + |  |  |
| **Glucocorticoid receptor (GR)** | + |  |  |
| **GLUT1** | + |  |  |
| **GLUT2** | - |  |  |
| **GLUT3** | + |  |  |
| **GLUT4** | + |  |  |
| **GMR alpha** | + |  |  |
| **GnIH (Rfrp)** | strong |  |  |
| **GnRH** | very weak |  |  |
| **GnRH Receptor** |  |  |  |
| **Gpr 120** | weak |  |  |
| **Gpr 39** | + |  |  |
| **Grp** | + |  |  |
| **GrpR** | + |  |  |
| **Ht1b (5-Ht1b)** | + |  |  |
| **Htr2a (5-Htr2a)** | + |  |  |
| **Htr2c** | - |  |  |
| **IGF** | + |  |  |
| **IGF1R** | + |  |  |
| **IGFBP-1** | - |  |  |
| **IL-1 Beta** |  |  |  |
| **Insulin I** | - |  |  |
| **Insulin II** | - |  |  |
| **Insulin Receptor** | + |  |  |
| **Insulin receptor substrate 2** | + |  |  |
| **Kir6.1** | - |  |  |
| **Kir6.2** | + |  |  |
| **Kiss** | - |  |  |
| **Kiss Receptor** | + |  |  |
| **Lep Receptor** | + |  |  |
| **Lipoprotein lipase (LPL)** | strong |  |  |
| **mc3R** | - |  |  |
| **mc4R** | - |  |  |
| **MCH** | + |  |  |
| **MCHR1** | + |  |  |
| **New-1** |  |  |  |
| **New-2** |  |  |  |
| **New-4** |  |  |  |
| **NMS (no.3)** |  |  |  |
| **Nmu** | + |  |  |
| **NPB** | + |  |  |
| **NPW** | + |  |  |
| **NPY** | + |  |  |
| **NPY-Y1** | N/A |  |  |
| **NPY-Y2** | N/A |  |  |
| **NPY-Y4** | N/A |  |  |
| **NPY-Y5** | N/A |  |  |
| **NSE** |  |  |  |
| **NT** | + |  |  |
| **NTR1** | - |  |  |
| **OprlR** | + |  |  |
| **orexin** | - |  |  |
| **orexin R1** | + |  |  |
| **orexin R2** | + |  |  |
| **Oxytocin** |  |  |  |
| **pAdcyap1** | - |  |  |
| **POMC** |  |  |  |
| **proGlu** | + |  |  |
| **proGlu (2007)** | - |  |  |
| **PTH2-R** | + |  |  |
| **resistin** | + |  |  |
| **Secretin** | strong |  |  |
| **SF-1** | + |  |  |
| **Sim1 (S1)** | - |  |  |
| **Socs-3** | + |  |  |
| **somatostatin (som)** | weak |  |  |
| **SSTY** |  |  |  |
| **STAT3** | + |  |  |
| **STAT5A** | + |  |  |
| **STAT5B** | + |  |  |
| **Sur1** | + |  |  |
| **Sur2** | + |  |  |
| **Syndecan 3** | strong |  |  |
| **T antigen** | + |  |  |
| **TAC2 (neurokinin B)** | weak |  |  |
| **TH** | + |  |  |
| **THRalpha** |  |  |  |
| **THRbeta** |  |  |  |
| **Tph** |  |  |  |
| **TRH** | - |  |  |
| **Ucn** | + |  |  |
| **Ucp2** | + |  |  |
| **VGF** | + |  |  |
| **VIP** | strong |  |  |
| **VIPR1** | + |  |  |
| **VIPR2** | + |  |  |
